# Comparison between SNP array and imputed data to estimate population structure and ROH hotspots in horse breeds

**DOI:** 10.1186/s12864-025-12256-8

**Published:** 2025-11-29

**Authors:** Giorgio Chessari, Paula Reich, Andrea Criscione, Clemens Falker-Gieske, Salvatore Mastrangelo, Serena Tumino, Salvatore Bordonaro, Donata Marletta, Jens Tetens

**Affiliations:** 1 Department of Agriculture, Food and Environment, University of Catania, Catania, 95131 Italy; 2 Department of Animal Sciences, Georg-August-University Göttingen, Göttingen, 37077 Germany; 3 Center for Integrated Breeding Research (CiBreed), Georg-August- University Göttingen, Göttingen, 37075 Germany; 4 Department of Agricultural, Food and Forestry Sciences, University of Palermo, Palermo, 90128 Italy

**Keywords:** Imputation, SNP, Whole-genome sequencing, Horse species, Genome diversity, Runs of homozygosity

## Abstract

**Background:**

Single nucleotide polymorphism (SNP) arrays are commonly used for studying the genomic structure and diversity of livestock breeds, but whole-genome sequencing (WGS) provides higher-resolution genomic data. Genotype imputation has become a standard practice for increasing the genomic resolution of association studies. This work aimed to extend imputation to biodiversity analyses, comparing SNP array data before and after imputation. A 40 k SNP dataset of 281 horses from 12 breeds (DS_SNP_) was imputed to sequence-level using a reference panel of 327 sequenced individuals, generating approximately 9 million markers after filtering (DS_IMP_). Both datasets were used to study genetic variability, population structure and runs of homozygosity (ROH).

**Results:**

Genetic indices and relationships showed similar trends for both datasets, with high Pearson correlations and Mantel test values (> 0.8) indicating that the imputed data are a reliable alternative to SNP array data for genetic studies. Multidimensional scaling and admixture analyses highlighted how the genetic proximity between breeds observed for the DS_SNP_ was amplified by the imputation process in cases of those breeds with a few sequences included in the WGS reference panel. ROH investigation showed overlapping homozygosity regions between the two datasets, highlighting the benefits of having more markers for gene and QTL annotation. Of the 141 ROH islands identified in the DS_SNP_, 79 overlapped perfectly with those found in the imputed data. Validation with the reference panel of 327 sequenced horses revealed a single ROH island on ECA11 shared across all three datasets, containing genes associated with morphology and behavioral traits.

**Conclusions:**

High correlations between SNP array and imputed data indicate that imputed genotypes provide a reliable alternative for assessing population structure and genetic diversity in horse breeds. Specifically, imputation can enhance the detection of ROH and the annotation of genes within ROH islands, with the reliability of these results depending on the quality of the reference panel and its representation of the studied breeds, among others.

**Supplementary Information:**

The online version contains supplementary material available at 10.1186/s12864-025-12256-8.

## Background

The study of genomic diversity in livestock is essential for conserving unique genetic resources, supporting breeding programs and safeguarding the cultural and economic heritage of local populations [1–3]. Horses show a remarkable diversity shaped by centuries of selective breeding, with some breeds maintaining well-defined characteristics [4, 5] and many others having more dynamic structures or facing challenges due to reduced census sizes [6–8]. Monitoring this diversity requires precise genomic tools capable of characterizing population structure and relationships among breeds, thereby informing conservation and selection strategies [9–11]. As in other livestock species, molecular approaches in horses have quickly evolved from microsatellites (STRs) [12–14] to medium- [15, 16] and high-density [17, 18] single nucleotide polymorphism (SNP) arrays, and more recently to whole-genome sequence (WGS) data [19, 20]. This technological progress has enabled increasingly precise characterization of equine genomic diversity [21].

While high-density genotype and WGS data can be advantageous for specific types of analyses compared to lower density SNP array data [22–24], especially the generation of WGS data is still expensive when applied to large sample sizes [25]. An affordable alternative to obtain sequence-level data is genotype imputation [26], which has become a key tool to increase marker density and resolution in genomic studies. It predicts untyped genotypes by leveraging haplotype information from high-density reference panels. The method exploits linkage disequilibrium (LD) patterns to infer missing alleles and reconstruct haplotypes shared between sequenced and genotyped individuals [27–29].

Genotype imputation is widely used to boost the statistical power of genome-wide association studies (GWAS) [e.g. 23, 30]. Increasing the marker density improves the resolution of GWAS and facilitates the discovery of quantitative trait loci (QTL) and candidate genes [31–33]. Although SNP array data are sufficient to map genomic regions associated with traits of interest, identifying the exact causal variants requires the availability of WGS data [24], which is also essential for applications beyond GWAS. Marker data capture only a small fraction of the total genomic variation, whereas WGS data encompass nearly all variants directly [34], a property that, with some limitations, also applies to imputed sequence data. However, the prediction of missing genotypes by imputation comes with a risk of errors [35], with its accuracy depending on several factors, such as reference panel size and composition, imputation software, SNP array density, and allele frequencies [15, 36, 37]. In follow-up studies, imputation accuracy can be assessed using metrics such as correlation coefficients, concordance rates, and software-specific measures like Beagle’s Dosage R-squared (*DR2*) [27, 38].

Imputation has been successfully applied to improve genomic prediction in several livestock species [39–41], and it is advantageous to facilitate meta-analyses by harmonizing the marker sets from different study cohorts [28]. Related to genetic diversity analyses, genotype imputation has been used to refine estimates of effective population size in sheep [42], detect runs of homozygosity (ROH) [43], and identify selection signatures in cattle [44]. However, apart from these few examples, it has not been investigated much in the context of biodiversity studies. In horses, research on imputation has focused chiefly on assessing its accuracy [15, 36, 37], while its application to biodiversity studies remains largely unexplored.

Given the limited research on genotype imputation in the equine species and its application in genetic diversity analyses, this paper aims to validate the use of imputed data for biodiversity studies in horses. Specifically, we (i) compare biodiversity estimates obtained from SNP array and imputed WGS data, and (ii) assess the potential of imputed genotypes for detailed analyses, such as the prediction of runs of homozygosity (ROH).

## Methods

### DNA sampling and whole-genome sequencing

From the 46 horses previously described in Criscione et al. [45], ten individuals (four Sanfratellano, three Purosangue Orientale Siciliano, and three Siciliano) were selected for whole-genome sequencing. We included the remaining 36 horses from the same study in the target dataset for imputation. Library preparation and next-generation sequencing of the samples were performed at Genomix4Life company (Salerno, Italy). Sequencing was conducted on an Illumina NovaSeq 6000 system, generating 150 bp paired-end reads at 20× genome coverage. We checked the quality of the FASTQ files using FastQC v0.12.1 (http://www.bioinformatics.babraham.ac.uk/projects/fastqc) and summarized the results with MultiQC v1.27.1 [46]: median per-base Phred scores were ≥ 32 for all positions, and no sequences were flagged as poor quality; adapter contamination was negligible, and the sequence length distribution was uniform, with most reads at the expected length.

### Construction of a WGS reference panel

The FASTQ files generated from sequencing were processed using the Genome Analysis Toolkit (GATK) v4.1.7.0 [47] following the GATK best practice recommendations [48, 49]. Briefly, *FastToSam* was used to convert FASTQ into uBAM format and to add the read group information. Sequencing adapters were marked with *MarkIlluminaAdapters* and reads were then aligned to the EquCab3.0 reference genome (GCA_002863925.1; Ensembl release 100) using *SamToFastq*, BWA-MEM v0.7.12 software [50] and *MergeBamAlignment*. After marking duplicate reads with *MarkDuplicates*, filtered BAM files were recalibrated on base quality scores (*BQSR*) using *BaseRecalibrator* and *ApplyBQSR*, incorporating known variant sites from the Ensembl dbSNP database (build 151, accessed via https://ftp.ensembl.org/pub/release-100/variation/vcf/equus_caballus/). Variant calling was performed per sample using *HaplotypeCaller* in ERC GVCF-mode. The resulting GVCF files were then merged into a single file using *CombineGVCFs*. Publicly available WGS data from 317 horses representing 46 global breeds, obtained from the European Nucleotide Archive (ENA) at EMBL-EBI and processed similarly in a previous study [36], were combined with the ten Sicilian samples. This resulted in a final dataset comprising 327 sequenced individuals. Finally, the combined GVCF files were jointly genotyped with *GenotypeGVCFs*. 

Raw SNPs were first hard-filtered using *VariantFiltration* with an ExcessHet threshold > 54.69. Subsequently, a soft-filtering step was applied via the *Variant Quality Score Recalibration* (*VQSR*). For this process, the Illumina Infinium EquineSNP50 BeadChip (54,602 markers) and the GeneSeek EquineSNP65 BeadChip (65,157 markers) were lifted over to the EquCab3.0 reference genome and used as truth datasets for *VariantRecalibrator* and *ApplyVQSR* tools. The liftover procedure utilized the SNPchiMP v3 repository [51] to convert Illumina SNP array IDs to dbSNP rsIDs, and EquCab3.0 positions were retrieved from the dbSNP build 151 using *SelectVariants*. Because two truth sets were employed, a transition/transversion (Ti/Tv) free recalibration method was applied, with a truth sensitivity threshold set to 99%. The final WGS reference dataset, which included 327 horses (see Additional file 1: Table S1) and 24,222,244 filtered SNPs, was phased using Beagle v5.1 [52] with an effective population size (*N*_*e*_) parameter of 1000.

### Description of SNP array data

A subset of breeds was selected from two medium-density SNP datasets and used for the analyses. Breeds were included only if they were represented in both the SNP array datasets and the WGS reference panel, with at least three individuals per breed in each dataset to ensure reliable imputation. More specifically, 36 Sicilian horses from Sanfratellano, Purosangue Orientale Siciliano and Siciliano breeds, genotyped using the Illumina Equine SNP70k BeadChip array (61,746 autosomal SNPs), were retrieved from Criscione et al. [45]. Additionally, 245 individuals from nine different breeds genotyped with the Illumina Equine SNP50k BeadChip array (40,861 autosomal SNPs) were obtained from Petersen et al. [16] (see Additional file 1: Table S1 for detailed dataset information). Both datasets were updated to the EquCab3.0 genome assembly (GCA_002863925.1; Ensembl release 100) to harmonize chromosome coordinates, SNP positions and rsIDs using Plink v1.9 [53]. The merged dataset, hereafter referred to as DS_SNP_, included 281 samples genotyped for 40,518 common markers mapped on autosomes (Table 1).

### Imputation of the SNP dataset

Markers in the DS_SNP_ were first made concordant with the WGS reference panel using conform-gt v24May16.cee [54] and then phased with Beagle v5.1 [52] with *N*_*e*_ set to 1000. Imputation to sequence-level resolution was performed using the established WGS reference panel and Beagle v5.1 [52] with the same *N*_*e*_ parameter, resulting in 23,386,994 imputed markers on autosomes. The quality of imputation was assessed using the *DR2* metric [55], which is the estimated squared correlation between the imputed and true genotype dosages, for each SNP. The overall mean *DR2* across all autosomal SNPs was calculated, as well as mean *DR2* per chromosome, to evaluate the imputation accuracy across the genome. Markers with a *DR2* below 0.6 were excluded to retain high-confidence variants. To illustrate the relationship between *DR2* and minor allele frequency (*MAF*), a scatterplot was generated using a stratified random sample of the imputed dataset, selecting 1000 variants per 0.01 *MAF* bin to ensure balanced representation across the allele frequency spectrum. A Locally Weighted Scatterplot Smoothing (LOWESS) curve was optimized via 5-fold cross-validation, testing values of 0.005, 0.05, 0.10, 0.15, and 0.20. The final imputed dataset (DS_IMP_) comprised 8,996,896 markers for the 281 individuals from the DS_SNP_ (Table 1).

**Table 1:** Breed names, codes, and sample size for the DS_SNP_, DS_IMP_ and DS_WGS_ datasets

Breed	Code	DS_SNP_	DS_IMP_	DS_WGS_
Purosangue Orientale Siciliano	ORI	9^a^	9	3^c^
Sanfratellano	SAN	13^a^	13	4^c^
Siciliano	SIC	14^a^	14	3^c^
German Warmblood	GER	15^b^	15	21^d^
Akhal Teke	AKT	19^b^	19	4^d^
Arabian horse	ARA	24^b^	24	23^d^
Franches-Montagnes	FRA	19^b^	19	31^d^
Icelandic horse	ICE	25^b^	25	3^d^
Shetland pony	SHE	27^b^	27	4^d^
Quarter horse	QUA	40^b^	40	22^d^
Standardbred	STA	40^b^	40	24^d^
Thoroughbred	THO	36^b^	36	45^d^

^a^Criscione et al. [45]

^b^Petersen et al. [16]

^c^This study

^d^Reich et al. [36]

### Quality control and diversity within-breed analyses

The DS_SNP_ and DS_IMP_ datasets of the 281 individuals underwent identical statistical analyses. Prior to further investigation, multiallelic SNPs were excluded from the imputed dataset using BCFtools v1.9 [56], retaining only biallelic SNPs. Plink v1.9 [53] was used to perform quality control, applying the following thresholds: *MAF* ≥ 0.01, SNP genotype call rate ≥ 0.98, and individual call rate ≥ 0.98. After filtering, 40,168 variants remained in DS_SNP_ and 8,235,507 in DS_IMP_. Marker density graphs were generated using the *CMplot* package [57] in R environment [58], setting a *bin.size* of 1,000,000 (see Additional file 2: Figure S1). Genetic diversity indices, including observed (*H*_O_) and expected (*H*_E_) heterozygosity, *MAF* and inbreeding coefficient (*F*_IS_), were calculated for each breed with Plink v1.9 [53]. Correlations (*r*_p_) between genetic diversity indices were assessed through Pearson correlation analysis [59].

### Genetic relationships and population structure

Population relationships and structure were assessed for both DS_SNP_ and DS_IMP_ after pruning SNPs in high LD using the *indep-pairwise* function in Plink v1.9 [53]. Pruning parameters were set to *50 5 0.2* for the DS_SNP_ [45] and *100 25 0.2* for the DS_IMP_ [60, 61]. This resulted in 18,416 biallelic markers retained for DS_SNP_, and 445,509 for DS_IMP_. To investigate genetic relationships among breeds, a multidimensional scaling (MDS) analysis was performed based on pairwise identity-by-state (IBS) distances between individuals using Plink v1.9 [53]. Likewise, genetic distances for the 281 horses were also calculated as 1-IBS in Plink v1.9 and visualized as a Neighbour-Joining tree using SplitsTree v6.3.20 [62]. Population differentiation was quantified by pairwise *F*_ST_ distances estimated with Arlequin v3.5.2.2 [63], which were visualized as a heatmap using the R package *ggplot2* [64]. Reynolds’ genetic distances were also computed and used to construct a Neighbour-Joining tree in SplitsTree v6.3.20 [62]. Finally, ancestry proportions were estimated via the unsupervised model-based clustering algorithm implemented in Admixture v1.3.0 [65]. The most likely number of ancestral populations (*K*) was determined through cross-validation by evaluating prediction errors across *K* values. The results from the structure analysis were visualized using the R package *BITE* v1.2.0008 [66]. The statistical correlation (*r*_m_) between genetic distance matrices derived from DS_SNP_ and DS_IMP_ was assessed using a Mantel test [67].

### Runs of homozygosity (ROH)

ROH were investigated using the sliding windows method implemented in the R package *detectRUNS* v0.9.6 [68] by setting the following common parameters for DS_SNP_ and DS_IMP_: no missing or opposite genotypes were allowed in a ROH or sliding window, the maximum gap between consecutive SNPs was set to 1,000 kb and the minimum ROH length to 100 kb. The threshold parameter, which indicates the minimum proportion of sliding windows in which a SNP must be classified as homozygous to be considered part of a ROH, was set to 0.05. For the DS_SNP_, the minimum number of SNPs included in a ROH was set to 20, considering a sliding window of 25 markers and a minimum SNP density of one SNP every 100 kb. Because the DS_IMP_ had a much higher SNP density (~ 8.2 M variants; ~0.28 kb spacing) than the DS_SNP_ (~ 40 k variants; ~56 kb spacing), ROH-calling parameters for DS_IMP_ were scaled accordingly: a sliding window of 500 SNPs (covering ~ 140 kb on average), a minimum of 50 SNPs per ROH, and a minimum SNP density of one SNP every 50 kb were used to ensure each ROH is supported by multiple loci and to avoid spurious short calls. ROH segments were classified into five length classes using the nomenclature proposed by Kirin et al. [69] and Ferenčaković et al. [70]: 1–2, 2–4, 4–8, 8–16 and >16 Mb. The following statistics were calculated for each individual and breed: the mean number of ROH per individual (*N*_ROH_), the average length of ROH in Mb per individual (*L*_ROH_), the population genomic inbreeding coefficient (*F*_ROH_), calculated for each individual as the ratio between the total length of the genome covered by ROH divided by the total autosomal genome length covered by SNPs (approx. 2.26 Gb for DS_SNP_ and 2.28 Gb for DS_IMP_), and the breed total length covered by ROH segments in Mb (*S*_ROH_). Correlations (*r*_p_) among ROH indices were evaluated using Pearson’s linear correlation model [59].

### ROH Islands

Highly recurrent ROH regions (ROH islands) were identified by converting the SNP incidence within ROH per population into standardized *z*-scores and calculating the corresponding *p*-values. Only the top 0.5% of SNPs was considered to define an island in the DS_SNP_ and DS_IMP_ datasets [3, 71, 72]. Since DS_IMP_ offers a higher marker resolution, the reliability of the results was validated by comparing the ROH islands identified through DS_IMP_ with those obtained from a high-density whole-genome sequencing dataset (DS_WGS_). The DS_WGS_ was generated by selecting the breeds under investigation from the WGS reference panel and extracting the list of quality-controlled markers. In total, the DS_WGS_ comprised 187 individuals and 8,235,507 biallelic markers (Table 1). ROH analysis was conducted using the same parameter settings as for the DS_IMP_, and ROH islands were identified following the same procedure. Finally, individual ROH islands were compared across all three different datasets (DS_SNP_, DS_IMP_ and DS_WGS_) to identify fully overlapping regions.

Markers within each ROH island were examined by variant effect prediction (VEP) using SnpEff v5.2c [73] with standard settings and *EquCab3.0.105* as the reference database to retrieve annotated gene lists. Variants were classified according to their predicted functional impact by SnpEff, which categorizes mutations as high, moderate, low, or modifier impact based on their potential effect on the encoded proteins. Only variants predicted to have a high or moderate impact were considered further to focus on those most likely to affect gene function. Moreover, QTL associated with markers within the genomic regions of interest were investigated in Animal QTLdb [74] implemented in the Genomic Annotation in Livestock for positional candidate LOci (GALLO) R package [75].

## Results

### Evaluation of genotype imputation

**Fig. 1:**
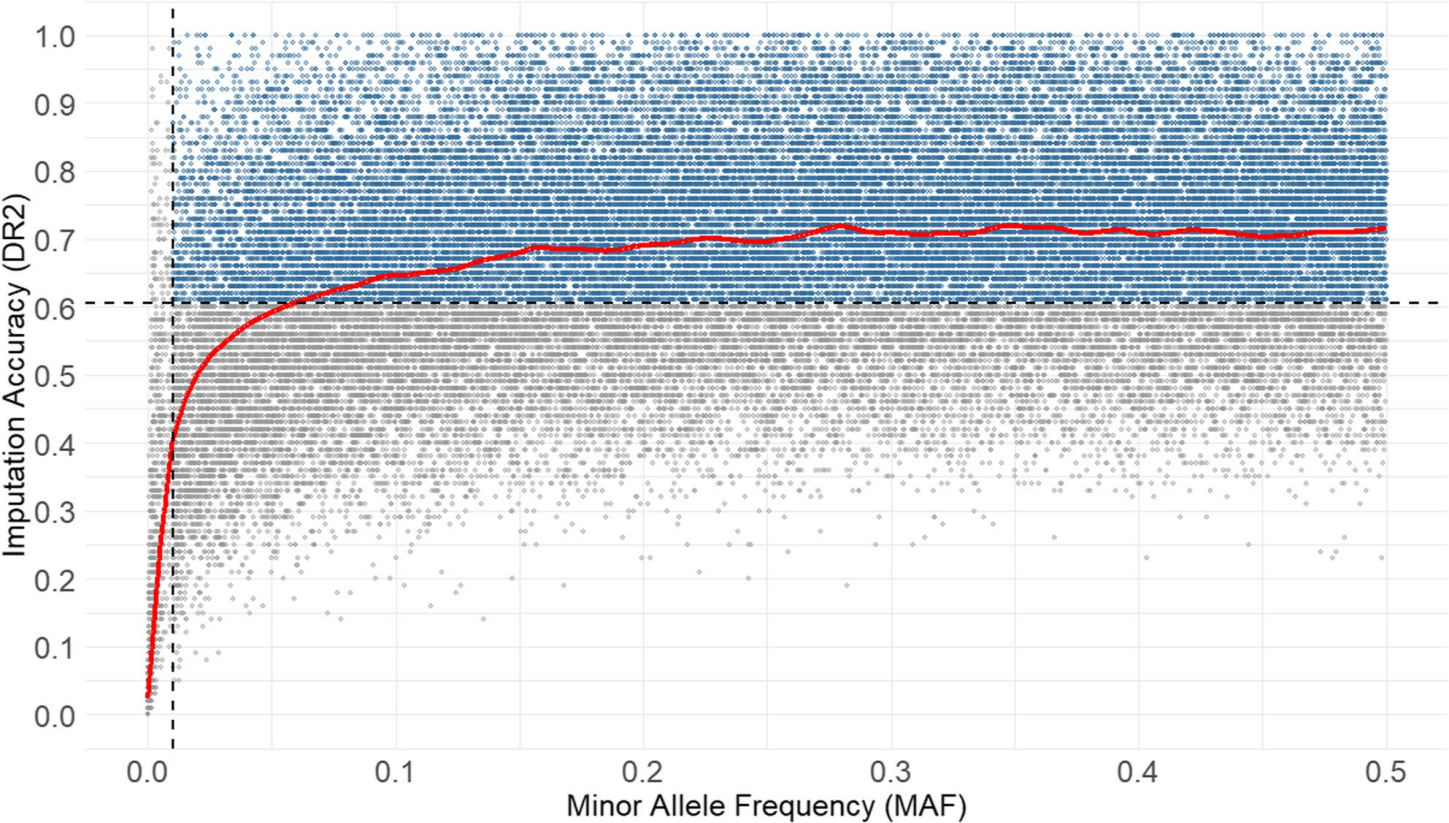
Scatterplot of *DR2* versus *MAF.* Scatterplot of imputation accuracy (*DR2*) versus minor allele frequency (*MAF*) for a stratified random sample (1000 variants per 0.01 *MAF* bin) from the imputed dataset. A red LOWESS curve (*f* = 0.05) highlights the overall trend. Dashed lines indicate the thresholds applied in downstream analyses (*DR2* > 0.6 and *MAF* ≥ 0.01)

The mean *DR2* values across all autosomes provided an overview of imputation quality, with limited variation among chromosomes. Values ranged from 0.42 on ECA12 to 0.50 on ECA17 (see Additional File 3: Table S2). These differences were not directly related to chromosome length, but in some cases reflected regions with low SNP coverage in the original array, as illustrated by the marker density plots (Additional File 2: Figure S1). Applying a commonly used threshold of *DR2* > 0.6 retained 38.5% of the imputed variants (Additional File 4: Figure S2), while increasing the overall mean *DR2* from 0.47 to 0.73. After filtering, the lowest mean value was observed for ECA31 (0.70; see Additional File 3: Table S2), indicating consistently high reliability across the genome for the variants included in downstream analyses. The relationship between imputation quality and allele frequency was further investigated using the scatterplot of *DR2* against *MAF*. A clear dependency was observed (Fig. 1): imputation accuracy remained stable for common variants (*MAF* > 0.20) but decreased sharply for rare variants (*MAF* < 0.05). The best fit for the LOWESS smoothing curve was obtained with *f* = 0.05 (CV-MSE = 0.02178), which most accurately captured the observed trend.

### Genetic diversity indices

Genetic diversity indices for both the DS_SNP_ and DS_IMP_ are reported in Table 2. All the imputed values of *H*_O_, *H*_E_ and *MAF* were on average lower than those of the DS_SNP_ by about 26%, but still showed the same trend. The analysis identified SIC, GER and QUA horses as the three breeds with the highest internal genetic variability. In contrast, the two light-draft breeds ICE and SHE had the lowest values. Notably, the ORI breed revealed very low *H*_E_ and *MAF* values, especially for the DS_IMP_. *H*_O_ was always higher than *H*_E_ within the same dataset, except for SHE, STA and ARA horses. Pearson correlation coefficients between the DS_SNP_ and DS_IMP_ were high across all genetic diversity indices, with *r*_p_ > 0.96 for *H*_O_ and *F*_IS_. All correlations were statistically significant (*p*-value < 0.001).

**Table 2:** Genetic diversity indices calculated for both DS_SNP_ and DS_IMP_

Breed	Dataset	H_O_ (SD)	H_E_ (SD)	MAF (SD)	F_IS_ (SD)
ORI	DS_SNP_	0.309 (0.241)	0.270 (0.188)	0.203 (0.163)	0.066 (0.052)
	DS_IMP_	0.223 (0.235)	0.196 (0.191)	0.143 (0.157)	0.082 (0.043)
SAN	DS_SNP_	0.310 (0.199)	0.296 (0.166)	0.218 (0.150)	0.064 (0.021)
	DS_IMP_	0.225 (0.201)	0.213 (0.177)	0.151 (0.147)	0.078 (0.018)
SIC	DS_SNP_	0.327 (0.184)	0.316 (0.153)	0.233 (0.144)	0.013 (0.048)
	DS_IMP_	0.233 (0.194)	0.224 (0.171)	0.158 (0.145)	0.041 (0.032)
GER	DS_SNP_	0.330 (0.190)	0.318 (0.160)	0.238 (0.149)	0.002 (0.018)
	DS_IMP_	0.236 (0.201)	0.227 (0.179)	0.162 (0.151)	0.031 (0.017)
AKT	DS_SNP_	0.304 (0.187)	0.298 (0.167)	0.221 (0.152)	0.082 (0.038)
	DS_IMP_	0.222 (0.195)	0.216 (0.179)	0.154 (0.150)	0.088 (0.029)
ARA	DS_SNP_	0.288 (0.184)	0.293 (0.172)	0.218 (0.155)	0.130 (0.059)
	DS_IMP_	0.214 (0.188)	0.217 (0.181)	0.156 (0.152)	0.120 (0.059)
FRA	DS_SNP_	0.286 (0.190)	0.279 (0.171)	0.204 (0.152)	0.137 (0.050)
	DS_IMP_	0.221 (0.191)	0.218 (0.178)	0.155 (0.149)	0.091 (0.045)
ICE	DS_SNP_	0.265 (0.191)	0.263 (0.178)	0.193 (0.155)	0.198 (0.032)
	DS_IMP_	0.210 (0.181)	0.207 (0.171)	0.145 (0.143)	0.139 (0.032)
SHE	DS_SNP_	0.240 (0.190)	0.244 (0.184)	0.179 (0.158)	0.274 (0.054)
	DS_IMP_	0.200 (0.183)	0.203 (0.178)	0.144 (0.148)	0.177 (0.053)
QUA	DS_SNP_	0.325 (0.161)	0.324 (0.151)	0.240 (0.143)	0.019 (0.033)
	DS_IMP_	0.234 (0.178)	0.233 (0.171)	0.164 (0.146)	0.040 (0.028)
STA	DS_SNP_	0.285 (0.178)	0.289 (0.171)	0.214 (0.154)	0.138 (0.038)
	DS_IMP_	0.220 (0.184)	0.224 (0.181)	0.161 (0.152)	0.096 (0.038)
THO	DS_SNP_	0.316 (0.186)	0.307 (0.171)	0.231 (0.155)	0.044 (0.031)
	DS_IMP_	0.223 (0.200)	0.216 (0.188)	0.158 (0.157)	0.084 (0.022)
Pearson	***r***_**p**_	**0.96**	**0.83**	**0.85**	**0.96**

Breed codes (explained in Table 1), observed (*H*_O_) and expected (*H*_E_) heterozygosity, minor allele frequency (*MAF*), inbreeding coefficient (*F*_IS_) and relative standard deviations (SD) for the DS_SNP_ and DS_IMP_. Linear Pearson correlation coefficients (*r*_p_) are reported for each index at the end of the table

### Population genetics analyses

**Fig. 2:**
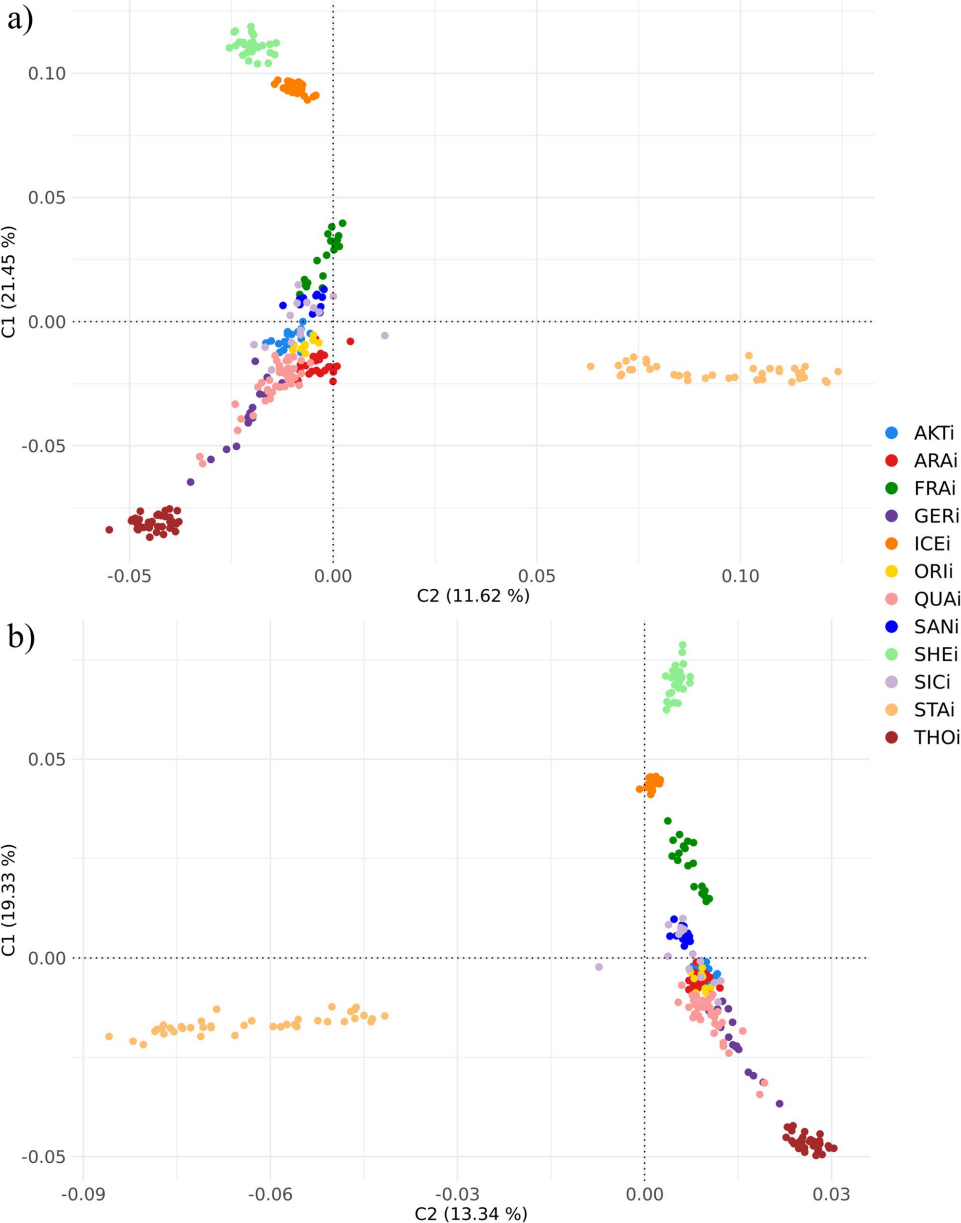
Multidimensional scaling plots for the (**a**)DS_SNP_and(**b**)DS_IMP_ . MDS analysis was performed on pruned datasets and based on pairwise identity-by-state (IBS) distances between 281 individuals

The first two components from MDS analysis (Fig. 2) on the pruned datasets explained 32.67% and 33.07% of the total variation in the case of DS_SNP_ and DS_IMP_, respectively.In both datasets, a clear separation between different horse breed groups was evident: light-draft horses represented by ICE and SHE clustered at the top of the plot, racing horses such as THO at the bottom, and riding horses (STA) on the right (DS_SNP_) or left (DS_IMP_) of the graph. All other breeds (saddle and riding horses) clustered together in the centre of the distribution, especially Sicilian (ORI, SIC and SAN) and endurance breeds (AKT and ARA). Some differences were observed between the DS_SNP_ and DS_IMP_ for the relative distance among the light and heavy draft horses. Specifically, in the DS_IMP_, FRA exhibited greater separation from riding horses, displayed an increased gradient of variability, and reduced its distance from light-draft horses. Additionally, a more pronounced overlap among ARA, AKT, and ORI breeds was evident.

The analysis of pairwise 1-IBS genetic distances, visualized through a Neighbour-Joining tree, showed a correlation of *r*_m_ = 0.81 (*p*-value < 0.01) in the Mantel test when comparing DS_SNP_ and DS_IMP_ matrices of individual distances. Some differences could be noticed, as presented in Additional file 5: Figure S3. The ASD tree for the DS_SNP_ dataset showed four main clades made up of draft breeds, oriental breeds, STA and saddle horses including THO, while DS_IMP_ revealed a clear isolated cluster also for SIC and SAN. Moreover, the orientation of STA changed after imputation, thus bringing it closer to the THO clade. In general, all the previously described groupings were maintained, and the influence of THO on GER became even more visible by their segmentation into two well-defined subgroups.

The results of the genetic distance matrices (*F*_ST_ and pairwise Reynolds’ genetic distances) calculated using the DS_SNP_ and DS_IMP_ (see Additional file 6: Figure S4 and Additional File 7: Figure S5) showed high concordance, with an *r*_m_ value of 0.96 (*p*-value < 0.01) in both cases. This suggests that both methods yield highly consistent results. The breeds were clustered in a similar manner for both datasets, with SHE being the most divergent, followed by ICE and THO for DS_SNP_, and THO and STA for DS_IMP_. In both cases, breeds were generally distinguished based on their attitude (light draft vs. racing vs. riding horses), confirming previous population results. Oriental breeds continued to cluster together, emphasizing the genetic similarity of ORI to other oriental horses rather than Sicilians. Similarly, GER and QUA were closely associated with the THO clade, demonstrating the significant influence of THO on the genetic makeup of these two breeds.

**Fig. 3:**
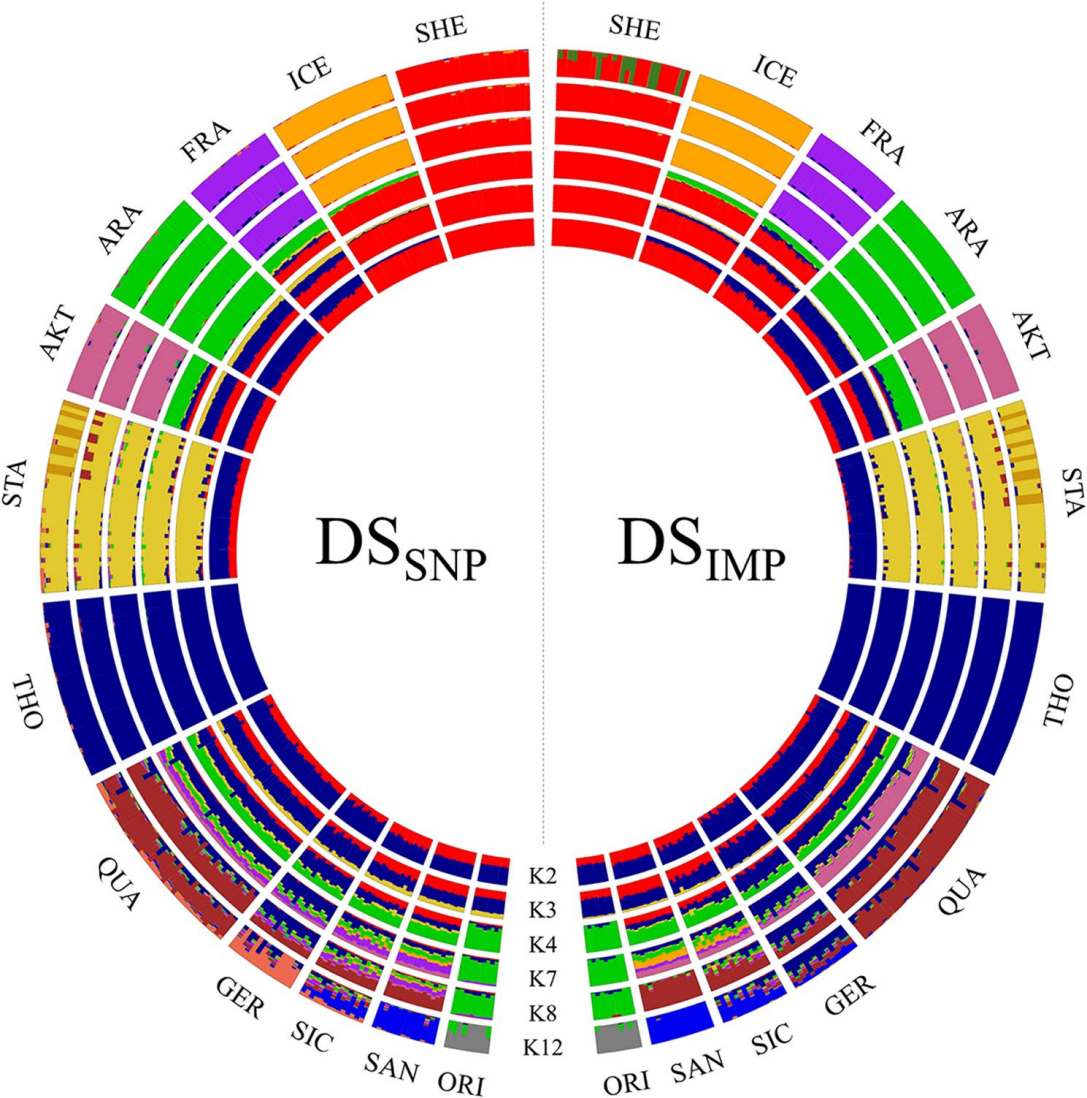
Circle plot of ancestral clusters for 12 different horse breeds. Ancestral clusters (*K*) inferred for 12 horse breeds in DS_SNP_ (on the left) and DS_IMP_ (on the right) datasets

Lastly, the admixture analysis investigating the population structure suggested *K* = 8 and *K* = 7 as the most likely number of ancestral clusters for the DS_SNP_ and the DS_IMP_, respectively (see Additional file 8: Figure S6). The circle plot in Fig. 3 summarizes the explanatory results of the admixture among breeds and the internal genetic variability. In particular, no significative differences were found between the two datasets. For the lowest *K* (*K* = 2), THO and SHE represented the two most divergent genetic patterns, while *K* = 3 confirmed the MDS distribution, assuming STA as a new, well-defined ancestral cluster. Increasing *K* to 4, the strong influence of the Arabian endurance horse (ARA) on AKT and Sicilian breeds, expecially on ORI, became clear. Both *K* = 7 and *K* = 8 clearly identified SHE, ICE, FRA, ARA, AKT, STA and THO as independent clusters, while a strong admixture was evident for the rest of the breeds. Finally, *K* = 12, which corresponds to the total number of breeds involved in the analyses, showed a difference in results between DS_SNP_ and DS_IMP_. Specifically, the two major differences included: (i) SHE, which displayed a homogeneous and distinct structure in DS_SNP_, but exhibited clear sub-structuring in DS_IMP_, and (ii) GER, which in DS_SNP_ demonstrated a unique genomic structure with partial admixture from THO, but in DS_IMP_ showed a pronounced and consistent influence from THO and QUA ancestry. As a result, SIC, which in DS_SNP_ reflected GER influence, lost this association in DS_IMP_. Notably, in both datasets, ORI remained entirely distinct from ARA.

### ROH investigation

**Fig. 4:**
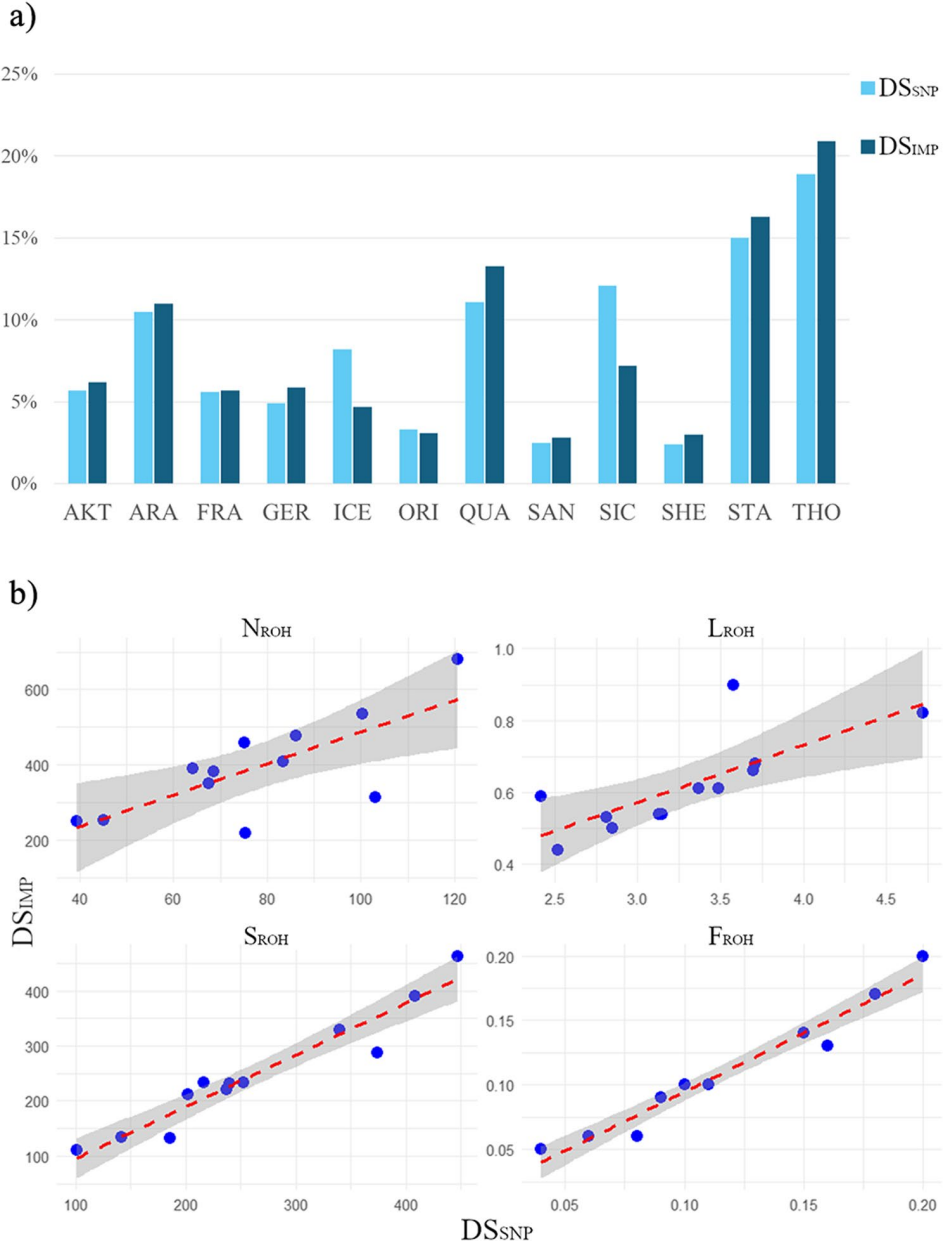
ROH summary statistics comparison. (**a**) Percentage contribution of each breed to the total amount of ROH for the DS_SNP_ and DS_IMP_. (**b**) Pearson correlation coefficients between DS_SNP_ and DS_IMP_ calculated for the four ROH parameters, also showing regression lines (dotted red) and confidential intervals (grey shadow)

 The ROH investigation revealed 23,026 and 117,040 ROH for the DS_SNP_ and DS_IMP_, respectively. Table 3 and Fig. 4 report the contribution of each breed to the total number of runs. For the same breeds, comparably large contributions were reported for the DS_SNP_ and DS_IMP_ analysis, with the exception for SIC and ICE, which showed a decrease in the number of ROH in the DS_IMP_. The mean number of ROH per individual (*N*_ROH_) showed similar trends, indicating THO and ARA as the breeds with highest values, while SAN and SIC had the lowest ones (see Additional file 9: Table S3). ECA1, the longest chromosome (~ 188.26 Mb), was the chromosome most affected by ROH in all cases. The *L*_ROH_ index for DS_SNP_ was 3.29 Mb on average, while the mean value for DS_IMP_ was 0.62. The DS_SNP_ analysis revealed that, across all breeds, 80% of the total amount of ROH were less than 4 Mb in length (ranging from 91% in ICE to 66% in STA), while almost all the ROH found in the DS_IMP_ analysis were shorter than 2 Mb (95% across all breeds). However, SIC was one of the breeds with the lowest *L*_ROH_ for both datasets (DS_SNP_ = 2.52, DS_IMP_ = 0.44). SIC was the breed with the lowest *F*_ROH_ value (DS_SNP_ = 0.04, DS_IMP_ = 0.05), while THO was the breed with one of the highest values (DS_SNP_ = DS_IMP_ = 0.20). The Pearson linear correlations between ROH indices calculated from SNP array data and imputed data were all high and statistically significant (*r*_p_ ranging from 0.74 to 0.98, *p*-value < 0.01) (Fig. 4). Notably, the strongest correlations were observed for *S*_ROH_ (*r*_p_ = 0.96, *p*-value < 10^− 6^) and *F*_ROH_ (*r*_p_ = 0.98, *p*-value < 10^− 6^), while *N*_ROH_ and *L*_ROH_ showed slightly lower but still substantial correlations of 0.74 and 0.76, respectively.

**Table 3:** Runs of homozygosity per breed and dataset

Breed	DS_SNP_		DS_IMP_	
	ROH	% total	ROH	% total
ORI	751	3.30%	3678	3.10%
SAN	586	2.50%	3261	2.80%
SIC	2782	12.10%	8452	7.20%
GER	1126	4.90%	6883	5.90%
AKT	1301	5.70%	7231	6.20%
ARA	2408	10.50%	12,839	11.00%
FRA	1283	5.60%	6629	5.70%
ICE	1884	8.20%	5466	4.70%
SHE	550	2.40%	3485	3.00%
QUA	2565	11.10%	15,601	13.30%
STA	3443	15.00%	19,039	16.30%
THO	4347	18.90%	24,476	20.90%

Number of runs of homozygosity (ROH) and contribution to the total number of ROH found (% total) calculated per breed and dataset. Breed codes are given in Table 1

### ROH islands

The top 0.5% of the SNP-within-ROH fixation included 4128 markers (2702 avoiding duplicates among breeds) for the DS_SNP_, resulting in 141 ROH islands across the 12 horse breeds. A total of 667,049 (494,259 avoiding duplicates among breeds) and 1,128,934 (940,968 avoiding duplicates among breeds) markers were included in the top 0.5% of the DS_IMP_ and DS_WGS_, respectively, identifying 504 and 1038 ROH islands. Detailed results are reported in Table 4.

**Table 4:** Top 0.5% of the SNP-within-ROH information for the different breeds and datasets

	AKT	ARA	FRA	GER	ICE	ORI	QUA	SAN	SHE	SIC	STA	THO
DS_SNP_												
N SNP	290	250	211	624	283	281	248	938	236	330	204	233
Min%	47.4	54.2	47.4	40.0	40.0	66.7	40.0	30.8	55.6	28.6	70.0	61.1
Max%	68.4	70.8	73.7	53.3	64.0	100.0	87.5	61.5	77.8	57.1	95.0	86.1
ROH_isl_	8	11	7	21	9	9	7	37	9	12	4	7
DS_IMP_												
N SNP	61,376	52,413	61,690	54,048	65,954	82,658	42,069	52,671	57,488	41,413	47,613	47,656
Min%	47.4	54.2	42.1	46.7	32.0	55.6	40.0	38.5	48.1	35.7	60.0	69.4
Max%	73.7	83.3	68.4	86.7	72.0	100.0	75.0	84.6	77.8	78.6	95.0	100.0
ROH_isl_	43	40	47	61	37	72	24	55	25	51	19	30
DS_WGS_												
N SNP	202,281	59,850	46,979	58,547	300,883	63,035	44,623	78,352	55,108	116,269	53,294	49,713
Min%	75.0	65.2	45.2	47.6	66.7	100.0	54.5	75.0	100.0	66.7	58.3	71.1
Max%	100.0	91.3	74.2	76.2	100.0	100.0	77.3	100.0	100.0	100.0	95.8	100.0
ROH_isl_	158	61	54	71	270	63	31	67	41	127	52	43

Top 0.5% of the SNP-within-ROH information for all breeds of the three datasets DS_SNP_, DS_IMP_ and DS_WGS_. Number of markers included in ROH islands (N SNP), minimum (Min%) and maximum (Max%) percentage of fixation explained by the top markers, and number of ROH islands detected (ROH_isl_). For a full definition of the acronyms see Table 1

SAN was the breed with the highest number of islands (37) in the DS_SNP_, ORI (72) in the DS_IMP_ and ICE (270) in the DS_WGS_. The number of single ROH islands shared across the datasets was 79 between DS_SNP_ and DS_IMP_, 152 between DS_IMP_ and DS_WGS_, and 43 between DS_SNP_ and DS_WGS_. Thirty-three single ROH islands were simultaneously reported across all three datasets, with a number of markers with predicted high or moderate impact on protein-coding sequences identified by VEP of 11 for DS_SNP_, 796 for DS_IMP_, and 534 for DS_WGS_ (see Additional file 10: Table S4). In case of shared ROH islands, the highest numbers were identified on ECA3 (between 35.14 Mb and 39.37 Mb and shared by nine breeds) and ECA11 (between 23.06 Mb and 36.06 Mb and shared by eight breeds) for the DS_SNP_. In contrast, ECA7 was the chromosome with the most shared ROH island for the DS_IMP_ (between 41.36 Mb and 76.83 Mb and shared by ten breeds) and DS_WGS_ (between 40.41 Mb and 44.28 Mb and shared by nine breeds). A list of the shared ROH islands is provided in Additional file 11: Table S5, Table S6 and Table S7, corresponding to DS_SNP_, DS_IMP_ and DS_WGS_, respectively, highlighting only those hotspots shared by at least six out of the 12 breeds within each dataset. Among these, DS_SNP_ exhibited a total of four ROH hotspots that completely overlapped with DS_IMP_, increasing the total number of SNPs within ROH from 503 to 74,892. Moreover, VEP analysis performed on these four regions showed three and 468 markers with a predicted high or moderate impact on protein-coding sequences for the DS_SNP_ and the DS_IMP_, respectively. Only one ROH island on ECA11 was simultaneously present in all three datasets, with a complete overlap between 24.00 Mb and 31.30 Mb and including the *TTLL6* gene. A more extensive description of gene and QTL annotations for these markers is available in Additional file 11: Table S5, Table S6 and Table S7.

## Discussion

Imputation has become standard practice to increase the marker density and thus potentially resolution of association studies [25, 32, 33]. This study focused on comparing biodiversity measures before and after imputation, and assessing the potential of imputed genotypes for downstream analyses, with a focus on ROH detection. WGS data of 327 horses belonging to worldwide breeds were used as the reference panel to impute medium-density SNP array data from 281 horses to sequence level. The methodological investigations were designed to analyse the suitability of imputated data for the study of genomic diversity, with particular attention to the pattern of ROH. The imputation process allowed us to greatly increase the number of markers, filling the gaps present in the original medium-density SNP array data. Beadchip arrays are typically designed to capture variations that are common within specific populations or breeds, which may overlook rare or population-specific variants [76]. In this view, imputation plays a critical role in reducing the ascertainment bias often linked to commercial SNP arrays, as it provides a more complete and accurate representation of genetic variation in the analysed meta-population [77].

### Evaluation of genotype imputation

Imputation in real scenarios may be challenging in terms of accuracy [15], which depends on several factors, including reference panel size, genetic similarity between reference and target populations, imputation software, SNP array density, and allele frequencies [26, 78–80]. The influence of these factors on imputation accuracy in horses was analysed in detail in a previous study [36] on the establishment and validation of a horse reference panel, which largely overlaps with the one used in the present study, and was therefore not investigated further here. Depending on the imputation scenario, the accuracy, calculated as the correlation between observed and imputed genotypes, ranged between 0.64 and 0.70 for imputation from medium density to sequence level using the software Beagle 5.1 [36], giving a good indication of the imputation accuracy achieved in the present study. In the absence of real sequence data to compare the imputed genotypes with, as was the case for the SNP-genotyped animals in the present study, measures internally derived from the imputation software can be used to evaluate the accuracy of imputation [27, 38]. Accordingly, *DR2*, which is the internal metric provided by Beagle, is a widely used measure of imputation accuracy [55] and can guide quality control by filtering out poorly imputed genotypes [81], with suggested *DR2* cut-offs ranging from 0.30 to 0.80 depending on species and study goals [82]. In our case, the overall mean *DR2* across autosomes was 0.47, but increased to 0.73 after applying a *DR2* > 0.60 threshold, indicating that the markers retained for downstream analyses had sufficiently high accuracy.

As shown in several other studies [26, 83–85], we observed a clear dependency of imputation accuracy on *MAF*: rare variants (*MAF* < 0.05) were imputed with lower accuracy, whereas common variants (*MAF* ≥ 0.30) showed higher mean *DR2* values. This pattern reflects the lower representation of rare alleles in the reference panel, which limits the reliability of genotype predictions for these variants [83, 86]. *MAF*-based filtering, therefore, excludes very rare variants that are more prone to imputation errors. This consideration is especially relevant for small or genetically diverse reference panels, and is consistent with previous findings in livestock species [87].

### Genetic diversity indices

The estimation of genetic diversity indices is an important preliminary step for the interpretation of diversity outcomes. The equine medium-density SNP array was based on the Thoroughbred reference genome with supplementary data from six other breeds [88], potentially introducing an upward bias in diversity indices for these breeds [16] and under-detecting unique variants in others. A higher coverage of markers (such as high-density arrays) and the design of arrays based on a higher number of breeds, as it was applied for the equine high-density SNP array [89], could provide more precise results. On the other hand, increasing the panel density, as in our DS_IMP_ case, could reduce the number of markers informative for heterozygosity, ultimately decreasing its values [90]. Therefore, pruning is not recommended for calculating diversity indices because it may eliminate important markers capturing the true genetic structure and signs of inbreeding through LD [91].

Overall, our results reflect the highest genetic variability for breeds with relatively recent origins (SAN and GER), admit constant introgression (ORI) or lack a real management program (SIC) [45, 92]. High values of inbreeding were observed for those breeds with a small census size, e.g. SHE [6], and breeds geographically isolated or affected by past bottleneck events, such as ICE [8]. An observed heterozygosity higher than the expected one can be related to a process of migration or introgression, as in the case of the ORI breed. It is well known that Arabian stallions are currently used in mating plans of this Sicilian breed and represent an important source of genetic flow, leading to an increase of the within-breed diversity [45]. On the contrary, an observed heterozygosity lower than the expected one can be explained by an increase of inbreeding, as in the case of SHE, a breed mainly reared on the Shetland islands (Scotland). This limited geographical area, combined with the small number of individuals (< 1000 registered horses) and the tendency to select stallions based on morphological characteristics (height at the withers or colour) rather than on pedigree information, increased the inbreeding rate [6], as confirmed by the highest *F*_IS_ estimate in the present study. Similar results were found by Petersen et al. [16], who analysed the same breeds (except for the Sicilian ones) using a pruned SNP dataset (~ 10 k markers), with *H*_E_ values ranging from 0.23 to 0.31, thus reflecting the results of our DS_SNP_ (0.24–0.32). With *H*_E_ values ranging from 0.20 to 0.23 in the DS_IMP_, deviations for both minimum and maximum values could be observed compared to the DS_SNP_. However, Pearson correlation coefficients between DS_SNP_ and DS_IMP_ were high for all calculated indices, suggesting that the imputation process had accurately predicted these genetic parameters.

### Population genetics

The data included in this study were collected from populations exhibiting significant phenotypic and attitude diversity. Several analytical approaches were used to calculate genetic distances, all of which produced similar results for the two datasets under investigation, as evidenced by the high correlation coefficients between the parameters calculated for them.

Notably, the lowest Mantel correlation was observed for the genetic distances calculated using 1-IBS, which were visualized through MDS and Neighbour-Joining tree plots, both of which calculate genetic similarity between individuals based on shared alleles. Specifically, MDS provides a spatial, distance-preserving view of genetic relationships, while the Neighbour-Joining tree offers a hierarchical, evolution-oriented representation [93]. These results suggest that, although there remains a strong correlation between the DS_SNP_ and DS_IMP_ data, this correlation is slightly less consistent than the breed-level comparisons made with *F*_ST_ and Reynolds distances, which are better suited for differentiating breeds based on their shared ancestry and for capturing population structure based on allele frequencies across populations.

As mentioned above, imputation accuracy tends to increase as the number of related individuals in the reference panel increases [36, 94]. For this reason, it can be assumed that the DS_IMP_ results are partially biased for those breeds with a limited number of sequenced individuals in the reference panel and derive from an imputation that exploits the information from genetically close breeds. MDS analysis supports this hypothesis well: in particular, a complete overlap between ARA (23 sequenced individuals), AKT and ORI (4 and 3 sequenced individuals, respectively) was observed in the case of the DS_IMP_, which was not the case for the results obtained from the DS_SNP_. The Arabian genetic lineage strongly influenced AKT and ORI breeds during their evolution, as corroborated in the present study by admixture analysis for K ≤ 3 and the unique clade in the Neighbour-Joining tree. Arabian horses have been introduced in ORI’s mating plan since 1864 to improve its endurance aptitude and agility [45, 95]; Kang et al. [96] explained how Arabian horses contributed the most to the ancestry of the Akhal-Teke horse using both SNP and mitochondrial DNA data.

As expected, breeds with a high number of sequences in the reference panel (THO, STA, QUA, FRA, ARA, GER) retained their own distribution in the two dimensions of the MDS plot. Despite the low number of sequences, the ICE and SHE tended to maintain their position in the imputed MDS plot, probably due to the low within-breed variability given their geographic isolation and strict morphological selection [6, 8]. The German Warmblood horses included in the reference panel represented a variety of German Warmblood populations (Hanoverian, Holsteiner, Oldenburger, Trakehner), considered a single strain, while the SNP data only included the Hanoverian breed. However, there did not appear to be any signs of bias in the results, highlighting the strong intermingling of these German breeds rather than their divergence.

### Runs of homozygosity

The study of ROH distribution has been used in many livestock species [97, 98] to unravel the homozygosity pattern and selection history of understudied populations [69, 99]. Sharing of ROH in specific regions of the genome between different individuals of the same breed or species (so-called ROH islands) can hint at the traces resulting from selective pressure, whether natural or artificial [100, 101]. In the present study, the investigation of ROH within the two datasets based on the same animals before and after imputation revealed differences mainly related to the number and the length of the detected ROH, probably due to the increased number of markers after imputation. Specifically, we observed that some ROH detected in DS_SNP_ were fragmented into multiple shorter islands in DS_IMP_, increasing the number of ROH and reducing their length within the same region.

The disadvantage of genotype imputation is the risk of potential errors resulting from the process, which can lead to potential false positive or false negative results in subsequent analyses [33, 35]. For this reason, the DS_WGS_ was used to validate the results of the ROH island analysis. To date, ROH investigations have commonly been performed using two methods of detection: sliding windows [102] and consecutive runs [103]. Even though the consecutive runs method seems to be the most powerful to avoid analytical bias [71, 104–106], the sliding windows method was the most used to detect ROH in horse species [20, 31, 101, 106]. In parallel with the development of these analyses, defining the correct parameter settings has always been challenging and is mainly influenced by the density of the SNP array used and the species under investigation [71, 90, 98]. Over the past years, some formulae were implemented to calculate the minimum number of SNPs that should be included in a ROH [107] and the minimum number of opposite SNPs allowed in a ROH [90]. However, the question of the best parameter settings remains unclear. Within the scope of the present study, many studies on ROH detection based on WGS or imputed data in animals and humans were compared [7, 19, 31, 99, 105, 108–111] to find the most suitable parameter settings. The relative contribution of each breed to the total number of ROH was similar for DS_SNP_ and DS_IMP_ [99, 112, 113], while the absolute number of ROH was higher for DS_IMP_ and therefore related to the density of the marker panel. Imputed or WGS panels offer greater marker resolution and higher sensitivity in detecting short runs, avoiding false long ROH [99, 114, 115], as confirmed in this study by *L*_ROH_ parameter estimation and ROH length classification, but they might not reveal consistent patterns [97, 115]. In a recent study, Falchi et al. [90] evaluated the effect of genotyping density on the detection of ROH and heterozygosity-rich regions (HRR) in a cattle breed using SNP arrays of different densities (50 k, 140 k and 800 k). The results demonstrated how high-density arrays can lead to an underestimation of long ROH (>8 Mb), while low-density arrays may lead to an overestimation of regions shorter than 4 Mb [70]. In the first case, the presence of opposite (heterozygous) markers breaks a long region into shorter ones, thus reducing the number of long runs, especially when the number of opposite SNPs allowed is set to 0, as in our analysis. This is particularly important in the case of imputed data, as imputation always comes with the risk of imputation errors [105], which increases the probability of false heterozygous markers. The outcomes from imputed data are consistent with those previously found in animals sequenced or genotyped with high-density SNP arrays that reported a high percentage of short runs (< 3 Mb) [44, 108, 116, 117]. In examining the correlation between DS_SNP_ vs. DS_IMP_, we found that although *N*_ROH_ and *L*_ROH_ had the lowest Pearson correlation coefficients, the values for *S*_ROH_ and *F*_ROH_ remained quite high.

### ROH Islands

ROH islands are defined as regions with continuous homozygosity and a fixation frequency above a certain threshold within a group of animals [72, 118]. When considering single breeds, SAN, ORI and ICE were the horses with the highest numbers of ROH islands in the DS_SNP_ (37), DS_IMP_ (72) and DS_WGS_ (270), respectively. In these cases, the high number of ROH islands, despite no signals of widely inbred status (low *N*_ROH_ and *F*_ROH_), suggests specific regions of the genome to be involvede in a consistent selection process for desirable traits.

Focusing on overlapping results across different datasets per breed revealed that 33 out of 141 ROH islands identified in DS_SNP_ were simultaneously detected in the top 0.5% across all scenarios, while 79 out of 144 ROH islands were confirmed within the top 0.5% of DS_IMP_, particularly in breeds with a high number of sequenced animals, such as STA, ARA, and FRA. Interestingly, the only shared ROH island that perfectly overlapped between the three datasets was located on ECA11 (from 25.2 Mb to 31.3 Mb). This homozygous hotspot on ECA11 has already been found in several studies in horses [45, 101, 119, 120] and has been linked to body size [121, 122], insect bite hypersensitivity [122], hair density and coat texture [92], and some biological processes such as embryonic skeletal system morphogenesis or cellular response to starvation [92, 101]. The importance of this region becomes more evident when considering the VEP annotations for the variants included in DS_IMP_ and DS_WGS_, revealing additional variants and genes that are not detectable using the medium-density SNP panel. This genomic region, which encompasses a highly gene-dense area, potentially suffered from a reduction in genetic variability due to ongoing selective pressure [122]. This gene-dense region includes several important genes, such as the *HOXB* gene group (*HOXB1*, *HOXB2*, *HOXB13*), which is part of the larger *HOX* gene family, which is crucial for the regulation of developmental processes in animals and has been under selection pressure in Europe during the Sassanid Persian expansion [106]. *HOXB* (*homeobox B cluster*) plays a critical role in determining the identity of different body segments during embryogenesis [123, 124]. In horses, the gene was found in Arabians, Exmoor ponies, Lipizzian and other breeds [120], as well as in four German breeds [92] and Franches-Montagnes [106]. *TTLL6* (*tubulin tyrosine ligase like 6*) was the only gene found commonly in all the datasets, which is involved in the post-translational modification of tubulin, affecting microtubule stability in humans [125–127]; to date no information is available on its role in livestock species. We identified *CACNA1G* (*calcium voltage-gated channel subunit alpha1 G*) and *STXBP4* (*syntaxin binding protein 4*) in the DS_IMP_, which are related to muscle and skeletal functions in horse species [128, 129]. High-impact variants further mapped to the *KIF2B* (*kinesin family member 2B*) gene in the imputed and sequenced data. *KIF2B* is known as a meiosis-related gene involved in the fertility of bulls [130–132] and body temperature adaptation in cattle [133]. Finally, several other studies have identified the *LASP1* (*LIM and SH3 domain protein 1*) gene as influencing body size in Noriker, Kurdish and Franches-Montagnes horses [106, 120, 121], although its location, between 23.30 Mb and 23.35 Mb, is just outside the region of our ROH island.

When examining the shared ROH islands recurrent in at least six of the 12 breeds within the meta-population, several hotspots were identified that align with findings reported in previous studies on horses, including those by Criscione et al. [45], Grilz-Seger et al. [101], Nolte et al. [92] and Gmel et al. [106]. In particular, the gene annotation for the DS_SNP_ identified *ENSECAG00000018058* on ECA3, *CIDEA* on ECA8 and *TTLL6* on ECA11. The *CIDEA* (*cell death inducing DFFA like effector A*) gene is part of a family of genes involved in lipid metabolism and energy homeostasis. It plays an important role both in livestock, where it has been reported to regulate heat stress in Thoroughbred horses reared for racing competitions [134] and increase milk fat synthesis in ketotic cows [135], and in humans, where it has been linked to body fat distribution and susceptibility to obesity [136, 137]. Furthermore, the four ROH islands shared between DS_SNP_ and DS_IMP_ demonstrate the increased marker density achieved through imputation, which in turn enhances the rate of gene annotation. For DS_IMP_, *PKN1*, *OR7D16*, *OR7G44*, *OR52AD3*, *ENSECAG00000024469* and *ENSECAG00000021649* were the genes affected by high-impact variants located within the ROH island on ECA7, and *MYOM1* on ECA8 and *KIF2B* on ECA11. The *PKN1* (*protein kinase N1*) gene encodes a serine/threonine protein kinase that is involved in various cellular processes, including the body’s immune response in goats [138]. Other highly affected genes included *NINJ2* on ECA6, *TMEM17* and *NRXN1* on ECA15, *LRRC63*, *ENSECAG00000035289* and *ENSECAG00000032604* on ECA17 for DS_IMP_, and *DMRT3* and *PLGRKT* on ECA23 for DS_WGS_. *DMRT3* (*doublesex and mab-3 related transcription factor 3*) plays an important role in the development of the nervous system and has been particularly studied in the context of locomotion and gait patterns in horses, especially in Nordic breeds (including Icelandic) and Standardbred trotters [139–143].

## Conclusions

This study evaluated the reliability of genotype imputation for biodiversity analyses in horse breeds, showing that imputed sequence-level data can provide accurate and robust estimates of population structure and diversity in livestock. Moreover, the higher marker density improved the resolution of homozygosity mapping, especially for short ROH segments that are often missed by SNP arrays. Noteworthy is the potential of imputed data for detailed analyses such as ROH islands detection and gene annotation, while also mitigating ascertainment bias by capturing a broader range of genetic variation. Hence, while medium-density SNP arrays remain a cost-effective resource for biodiversity studies, genotype imputation thus represents a cost-efficient strategy to enrich SNP chip data and extend genomic analyses in horse populations and, more broadly, in livestock species. Future efforts to expand resequencing in local populations will be crucial to further improve reference panels and strengthen imputation-based approaches. Overall, combining SNP arrays with imputation offers a practical framework for biodiversity assessments and predictive genomic studies, balancing affordability with improved analytical resolution.

## Supplementary Information

Additional File 1. Table S1. Dataset composition for the WGS reference panel and SNP datasets. Description: Breed name and number of animals are reported. Geographic origin and region of sampling are also shown for SNP datasets.

Additional File 2. Figure S1. Marker density plot for the (a) DS_SNP_ and (b) DS_IMP_. Description: The DS_SNP_ dataset included 40,168 filtered variants, whereas the imputed DS_IMP_ dataset retained 8,235,507 biallelic SNPs after quality control and distributed across autosomes.

Additional File 3. Table S2. Mean *DR2* values by chromosome before and after filtering. Description: *DR2* values per chromosome (ECA) are shown for the raw dataset (all imputed markers) and after applying a *DR2* threshold of 0.6 to retain high-confidence markers. The last row indicates the genome-wide mean for each column.

Additional File 4. Figure S2*. DR2* values across all imputed markers. Description: The histogram shows the distribution of *DR2* values. Grey bars represent markers with *DR2* ≤ 0.6, while blue bars indicate markers with *DR2* > 0.6. The red dashed line marks the *DR2* threshold of 0.6 used to retain high-confidence imputed markers.

Additional File 5. Figure S3. Neighbor-Joining tree for 281 individuals. Description: The analysis was based on pairwise genetic distances (calculated as 1-IBS) between individuals from 12 horse breeds for the (a) DS_SNP_ and (b) DS_IMP_.

Additional File 6. Figure S4. Pairwise *F*_ST_ values estimated between the 12 horse breeds. Description: The lower diagonal refers to the DS_SNP_, while the upper diagonal to the DS_IMP_ dataset. 

Additional File 7. Figure S5. Neighbor-Joining tree for the 12 worldwide horse breeds. Description: The analysis was based on Reynolds’ pairwise genetic distances among the 12 horse breeds for the (a) DS_SNP_ and (b) DS_IMP_.

Additional File 8. Figure S6. Cross-validation errors across *K*s in Admixture analysis. Description: Distribution of the cross-validation errors (CV) (y-axis) of each inferred K genomic cluster (x-axis) in the admixture analysis, referring to the (a) DS_SNP_ and (b) DS_IMP_.

Additional File 9. Table S3. ROH summary statistics reported for breed and dataset. Description: The mean number of ROH per individual (*N*_ROH_), the average length of ROH in Mb per individual (*L*_ROH_), the population genomic inbreeding coefficient (*F*_ROH_), and the breed total length covered by ROH segments in Mb (*S*_ROH_). Standard deviation for each value is reported as well (*SD*).

Additional File 10. Table S4. Overlapping ROH islands between the top 0.5% of the three datasets within each breed. Description: Start and end position for each region are reported in Mb. The total number of markers within-island (M) and VEP results are reported, such as the number of markers filtered for high or moderate impact (F), as well as for the resulting gene (G) and QTL (Q) annotations.

Additional File 11. Table S5. VEP of the shared ROH islands for the DS_SNP._ Description: ROH islands shared among at least six breeds are shown, reporting coordinates (chromosome, start and end positions) and acronyms of the breeds involved. Moreover, the number of markers filtered with high or moderate impact is also shown, as well as for the resulting gene and QTL annotations. Genes related to markers with high impact are marked in red. Additional File 11. Table S6. VEP of the shared ROH islands for the DS_IMP_. Description: ROH islands shared among at least six breeds are shown, reporting coordinates (chromosome, start and end positions) and acronyms of the breeds involved. Moreover, the number of markers filtered with high or moderate impact is also shown, as well as for the resulting gene and QTL annotations. Genes related to markers with high impact are marked in red. Additional File 11. Table S7. VEP of the shared ROH islands for the DS_WGS._ Description: ROH islands shared among at least six breeds are shown, reporting coordinates (chromosome, start and end positions) and acronyms of the breeds involved. Moreover, the number of markers filtered with high or moderate impact is also shown, as well as for the resulting gene and QTL annotations. Genes related to markers with high impact are marked in red.

## Data Availability

Publicly available WGS data from 317 horses were acquired from the European Nucleotide Archive (ENA) at EMBL-EBI (https://www.ebi.ac.uk/ena/browser/home). The horse genome assembly EquCab3.0 (Accession number: GCA_002863925.1; Ensembl release: 100) and dbSNP (build 151 - accessed on September 2023) were retrieved from Ensembl database (https://www.ensembl.org/index.html). SNP data of the 36 Sicilian horses genotyped with Illumina Equine SNP70k BeadChip array were obtained from [45], while the other 245 individuals genotyped with Illumina Equine SNP50k BeadChip array were retrieved from [16] (detailed information in Additional file 1: Table S1). Finally, the WGS data of the 10 Sicilian horses used and analysed during the current study are available from the corresponding author on reasonable request.
