# Supplementary material for: Comparison between SNP array and imputed data to estimate population structure and ROH hotspots in horse breeds
Source: BMC Genomics. 2025 Nov 29;26:1086. doi: 10.1186/s12864-025-12256-8 (PMC12670763; doi:10.1186/s12864-025-12256-8)
Supplement: Supplementary file 1 — Additional File 1. Table S1. Dataset composition for the WGS reference panel and SNP datasets. Description: Breed name and number of animals are reported. Geographic origin and region of sampling are also shown for SNP datasets. [file 12864_2025_12256_MOESM1_ESM.docx]

| Breed | Number | Geographic origin | Region of sampling |
| --- | --- | --- | --- |
| WGS reference panel (Reich *et al.* 2022): Total of 327 individuals | | | |
| Akhal-Teke | 4 | Not reported | Not reported |
| American Bashkir Curly Horse | 2 | Not reported | Not reported |
| American Miniature Horse | 1 | Not reported | Not reported |
| American Paint Horse | 4 | Not reported | Not reported |
| Arabian | 23 | Not reported | Not reported |
| Connemara Pony | 4 | Not reported | Not reported |
| Crossbred (Warmblood/Quarter Horse) | 2 | Not reported | Not reported |
| Curly Horse | 3 | Not reported | Not reported |
| Deutsches Reitpony | 1 | Not reported | Not reported |
| Duelmener | 1 | Not reported | Not reported |
| Franches-Montagnes | 31 | Not reported | Not reported |
| Friesian Horse | 7 | Not reported | Not reported |
| German Riding Pony | 2 | Not reported | Not reported |
| German Warmblood | 12 | Not reported | Not reported |
| Haflinger | 10 | Not reported | Not reported |
| Hanoverian | 6 | Not reported | Not reported |
| Holsteiner | 5 | Not reported | Not reported |
| Icelandic | 3 | Not reported | Not reported |
| Italian Trotter | 1 | Not reported | Not reported |
| Jeju Pony | 6 | Not reported | Not reported |
| Koninklijk Warmbloed Paard Nederland | 1 | Not reported | Not reported |
| Lipizzan Horse | 4 | Not reported | Not reported |
| Lowland (Erlunchun) | 7 | Not reported | Not reported |
| Lowland (inner Mongolia) | 7 | Not reported | Not reported |
| Lusitano | 1 | Not reported | Not reported |
| Mangalarga Marchador Horse | 1 | Not reported | Not reported |
| Marwari Horse | 1 | Not reported | Not reported |
| Mongolian | 2 | Not reported | Not reported |
| Morgan | 1 | Not reported | Not reported |
| Not specified (NA) | 10 | Not reported | Not reported |
| Native Mongolian Chakouyi Horse | 1 | Not reported | Not reported |
| Noriker | 1 | Not reported | Not reported |
| Norwegian Fjord | 1 | Not reported | Not reported |
| Oldenburger | 1 | Not reported | Not reported |
| Percheron | 1 | Not reported | Not reported |
| Quarter Horse | 22 | Not reported | Not reported |
| Saxon-Thuringian Heavy Warmblood | 1 | Not reported | Not reported |
| Shetland Pony | 4 | Not reported | Not reported |
| Shire | 1 | Not reported | Not reported |
| Sorraia | 2 | Not reported | Not reported |
| Standardbred | 24 | Not reported | Not reported |
| Swiss Warmblood | 2 | Not reported | Not reported |
| Tennessee Walking Horse | 1 | Not reported | Not reported |
| Thoroughbred | 45 | Not reported | Not reported |
| Tibetan | 31 | Not reported | Not reported |
| Traber | 3 | Not reported | Not reported |
| Trakehner | 1 | Not reported | Not reported |
| Vollblut | 1 | Not reported | Not reported |
| Welsh Pony | 2 | Not reported | Not reported |
| Yakutian Horse | 9 | Not reported | Not reported |
| Purosangue Orientale Siciliano | 3 | Not reported | Not reported |
| Sanfratellano | 4 | Not reported | Not reported |
| Siciliano | 3 | Not reported | Not reported |
| Sicilian SNP dataset (Criscione *et al.* 2022): Total of 36 individuals | | | |
| Purosangue Orientale Siciliano | 9 | Italy | Italy |
| Sanfratellano | 13 | Italy | Italy |
| Siciliano | 14 | Italy | Italy |
| Worldwide SNP dataset (Petersen *et al.* 2013): Total of 795 individuals | | | |
| Akhal-Teke | 19 | Turkmenistan | US and Russia |
| Andalusian | 18 | Spain | United States |
| Arabian | 24 | Middle East | United States |
| Belgian | 30 | Belgium | United States |
| Caspian | 18 | Persia | United States |
| Clydesdale | 24 | Scotland | US and UK |
| Exmoor | 24 | Great Britain | United Kingdom |
| Fell Pony | 21 | England | United Kingdom |
| Finnhorse | 27 | Finland | Finland |
| Florida Cracker | 7 | United States | United States |
| Franches-Montagnes | 19 | Switzerland | Switzerland |
| French Trotter | 17 | France | France |
| Hanoverian | 15 | Germany | Germany |
| Icelandic | 25 | Iceland | Sweden |
| Lusitano | 24 | Portugal | Portugal |
| Mandalarga Paulista | 15 | Brazil | Brazil |
| Maremmano | 24 | Italy | Italy |
| American Miniature Horse | 21 | United States | United States |
| Mongolian | 19 | Mongolia | Mongolia |
| Morgan | 40 | United States | United States |
| New Forest Pony | 15 | England | United Kingdom |
| North Swedish Horse | 19 | Sweden | Sweden |
| Norwegian Fjord | 21 | Norway | Norway |
| American Paint Horse | 25 | United States | United States |
| Percheron | 23 | France | United States |
| Peruvian Paso | 21 | Peru | United States |
| Puerto Rican Paso Fino | 20 | Puerto Rico | Puerto Rico |
| Quarter Horse | 40 | United States | United States |
| Saddlebred | 25 | United States | United States |
| Shetland Pony | 27 | Scotland | Sweden |
| Shire | 23 | England | United States |
| Standardbred | 25 | United States | Norway |
| Standardbred | 15 | United States | United States |
| Swiss Warmblood | 14 | Switzerland | Switzerland |
| Thoroughbred | 19 | England | UK and Ireland |
| Thoroughbred | 17 | England | United States |
| Tuva | 15 | Siberia | Russia |
