# Supplementary figures and images for: Comparison between SNP array and imputed data to estimate population structure and ROH hotspots in horse breeds
Source: BMC Genomics. 2025 Nov 29;26:1086. doi: 10.1186/s12864-025-12256-8 (PMC12670763; doi:10.1186/s12864-025-12256-8)

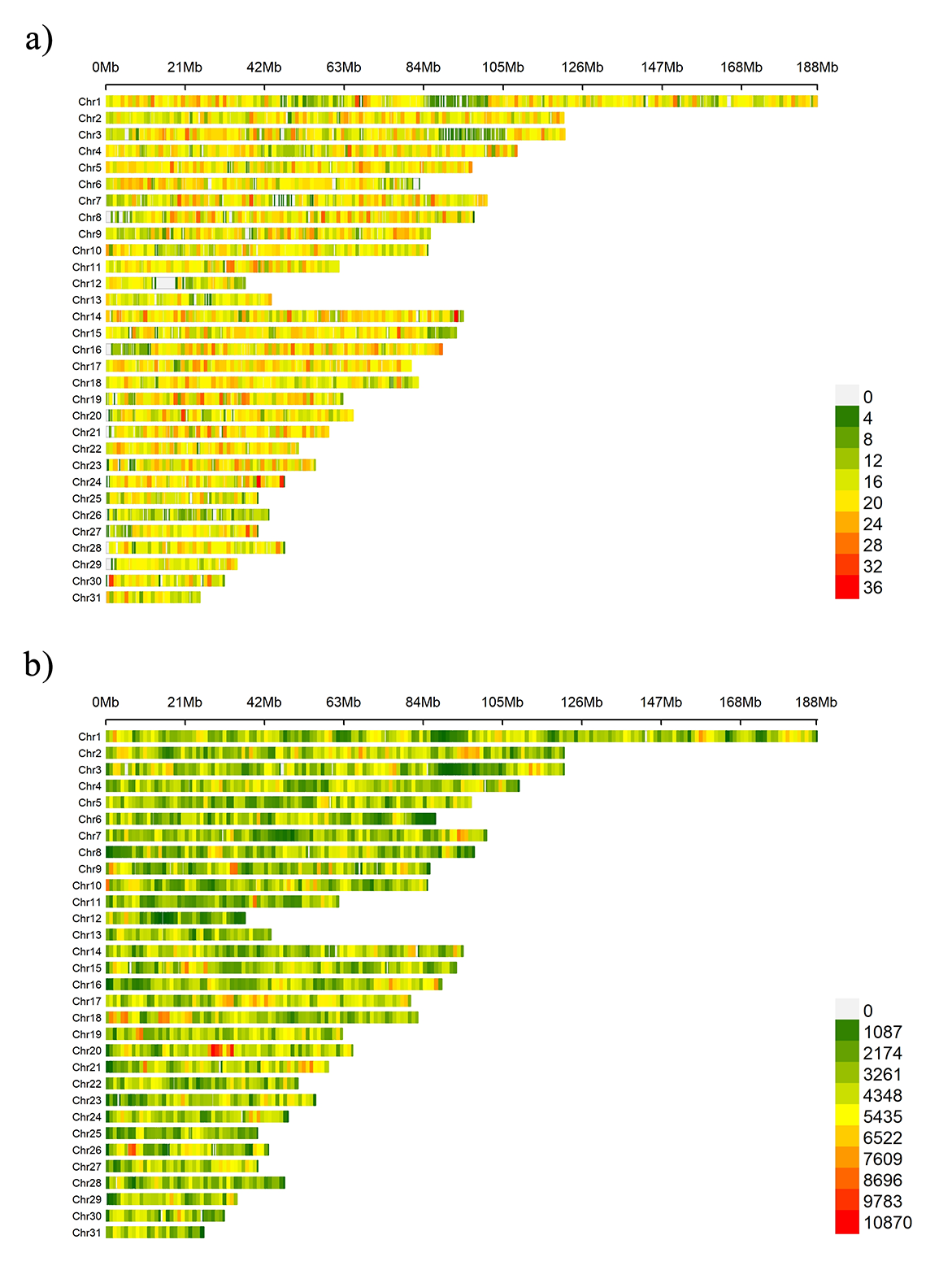

Supplement: Supplementary file 2 — Additional File 2. Figure S1. Marker density plot for the (a) DSSNP and (b) DSIMP. Description: The DSSNP dataset included 40,168 filtered variants, whereas the imputed DSIMP dataset retained 8,235,507 biallelic SNPs after quality control and distributed across autosomes. [file 12864_2025_12256_MOESM2_ESM.tif]

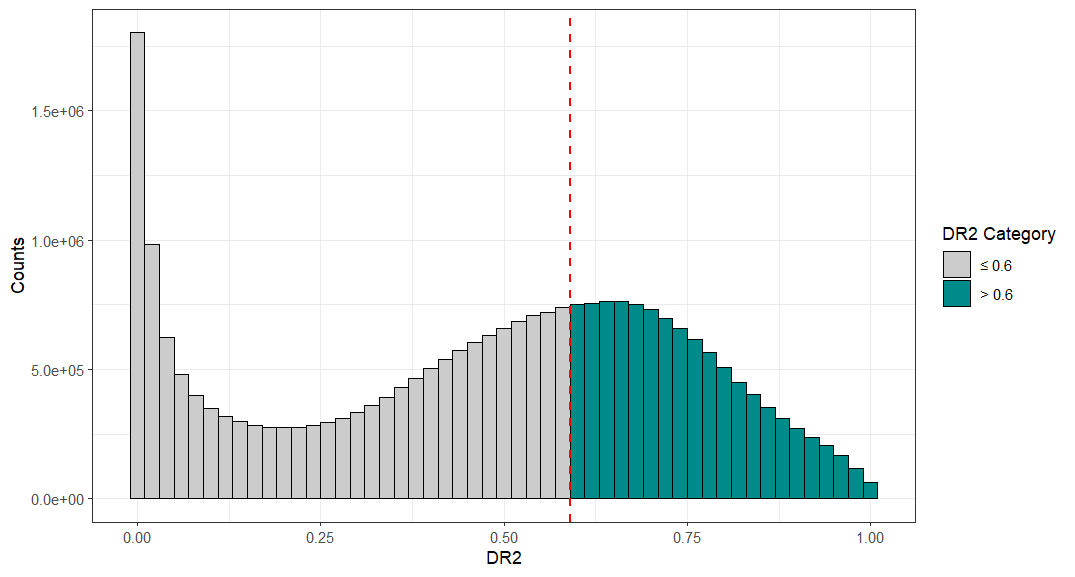

Supplement: Supplementary file 4 — Additional File 4. Figure S2. DR2 values across all imputed markers. Description: The histogram shows the distribution of DR2 values. Grey bars represent markers with DR2 ≤ 0.6, while blue bars indicate markers with DR2 > 0.6. The red dashed line marks the DR2 threshold of 0.6 used to retain high-confidence imputed markers. [file 12864_2025_12256_MOESM4_ESM.tiff]

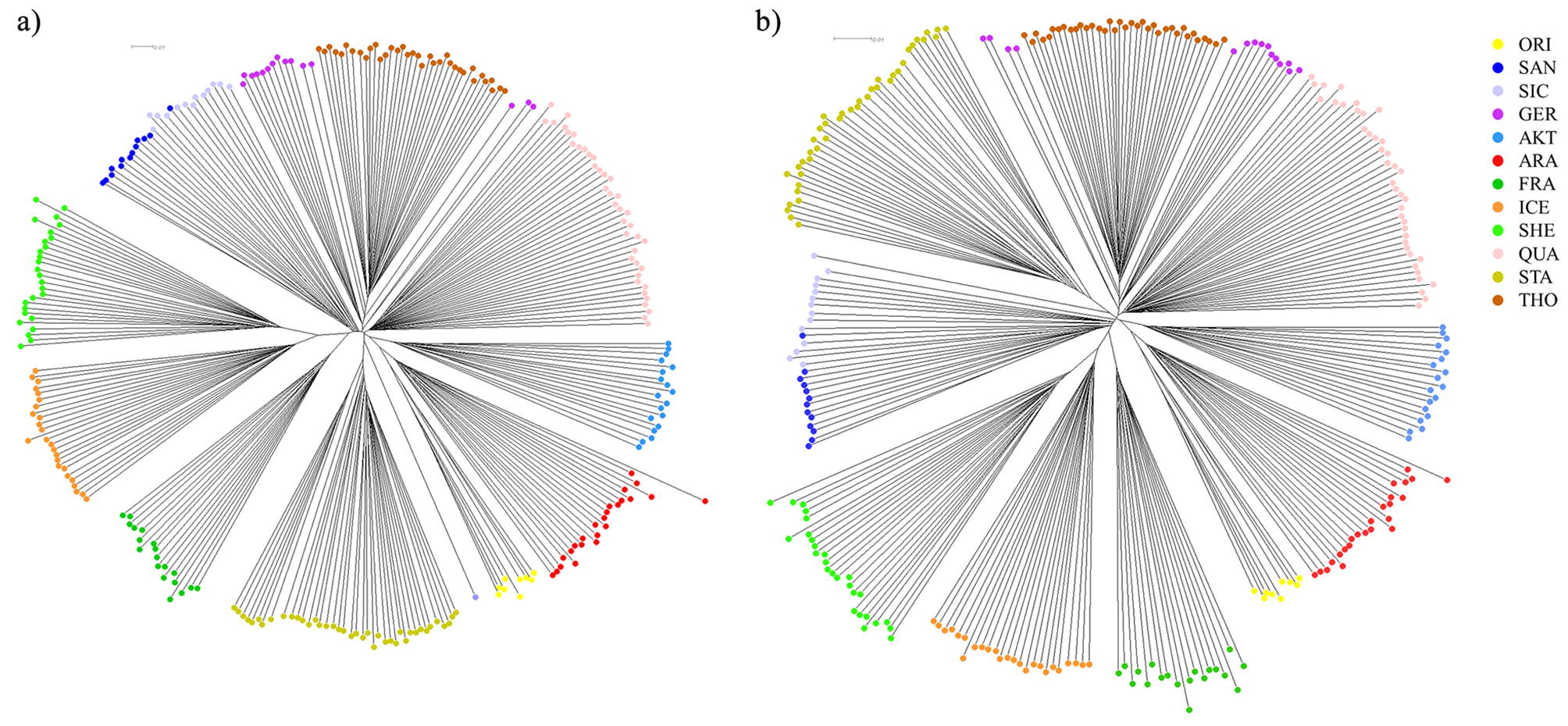

Supplement: Supplementary file 5 — Additional File 5. Figure S3. Neighbor-Joining tree for 281 individuals. Description: The analysis was based on pairwise genetic distances (calculated as 1-IBS) between individuals from 12 horse breeds for the (a) DSSNP and (b) DSIMP. [file 12864_2025_12256_MOESM5_ESM.tif]

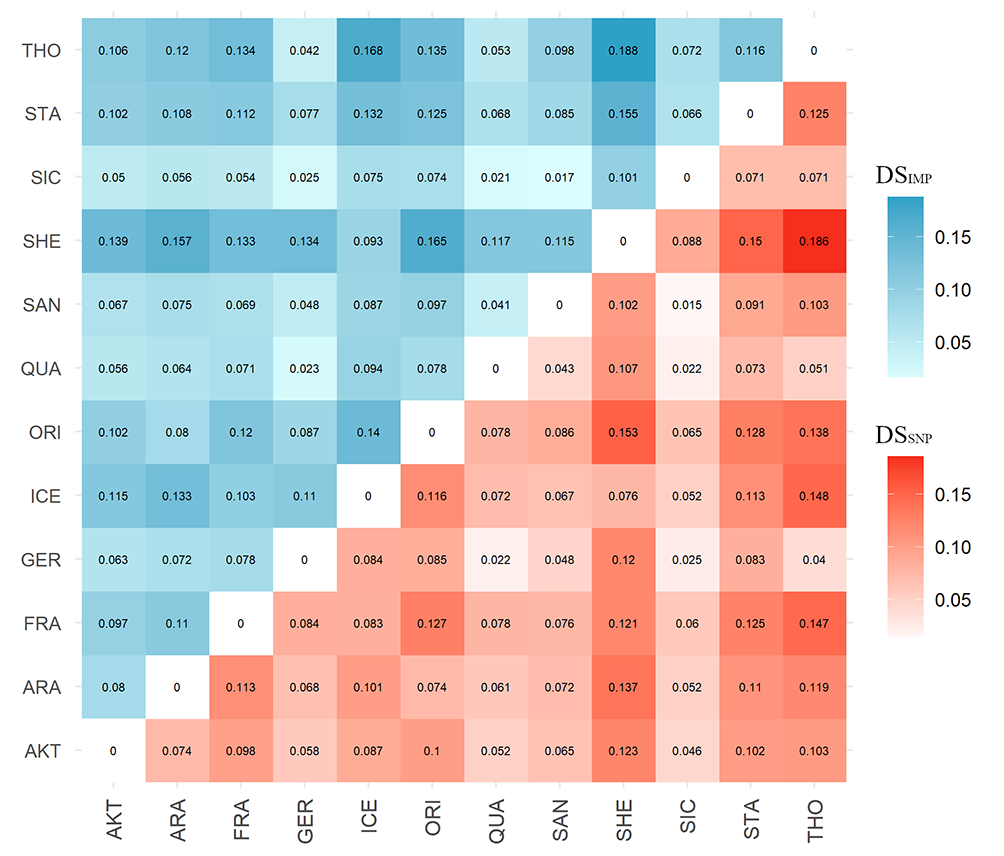

Supplement: Supplementary file 6 — Additional File 6. Figure S4. Pairwise FST values estimated between the 12 horse breeds. Description: The lower diagonal refers to the DSSNP, while the upper diagonal to the DSIMP dataset. [file 12864_2025_12256_MOESM6_ESM.tif]

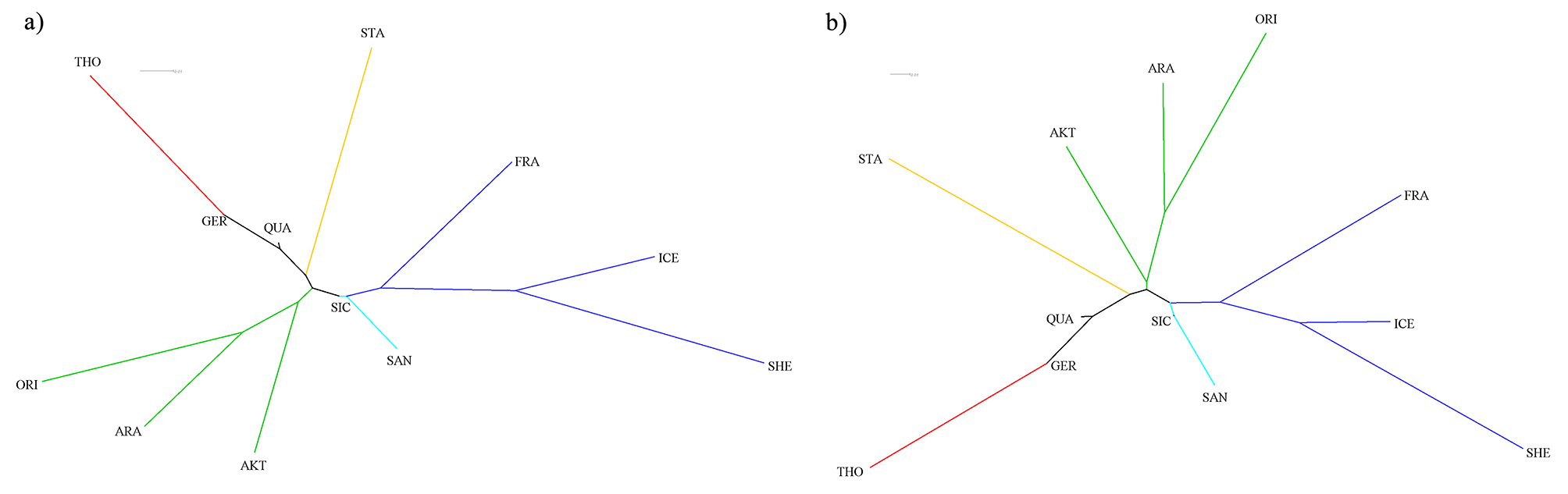

Supplement: Supplementary file 7 — Additional File 7. Figure S5. Neighbor-Joining tree for the 12 worldwide horse breeds. Description: The analysis was based on Reynolds’ pairwise genetic distances among the 12 horse breeds for the (a) DSSNP and (b) DSIMP. [file 12864_2025_12256_MOESM7_ESM.tif]

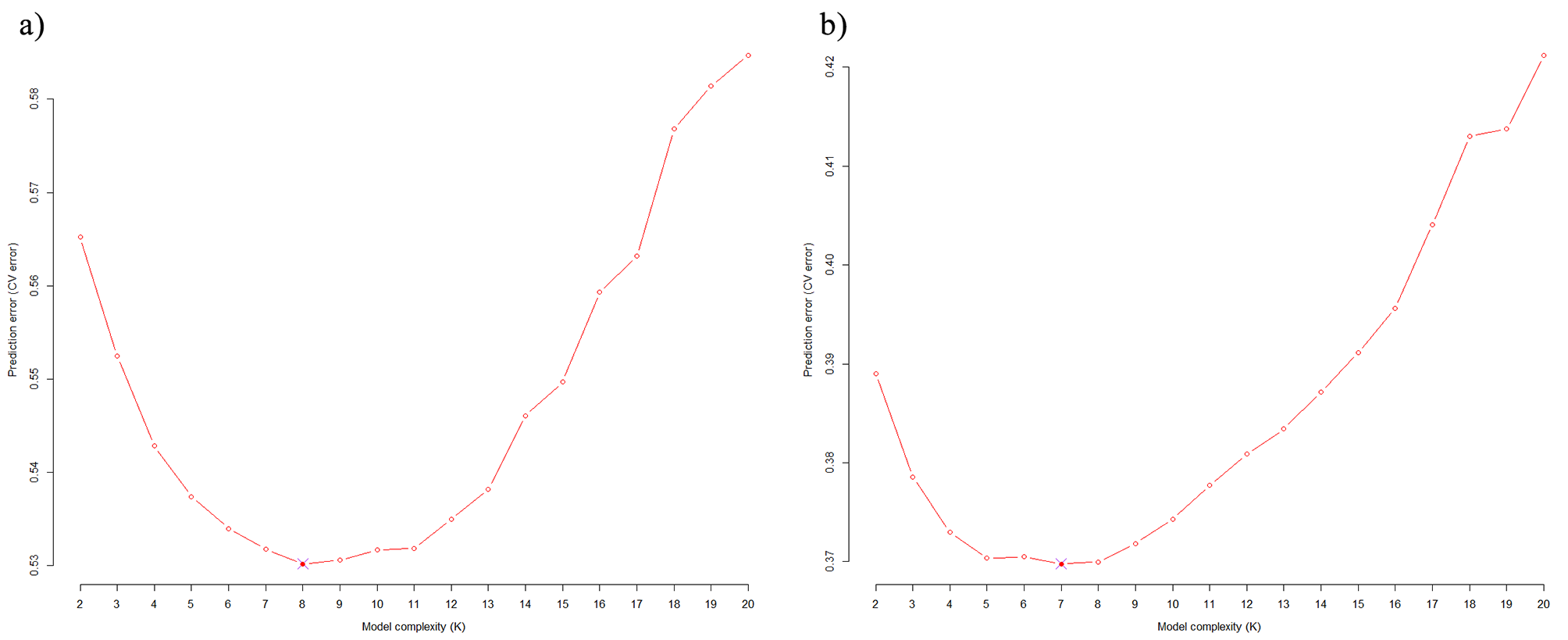

Supplement: Supplementary file 8 — Additional File 8. Figure S6. Cross-validation errors across Ks in Admixture analysis. Description: Distribution of the cross-validation errors (CV) (y-axis) of each inferred K genomic cluster (x-axis) in the admixture analysis, referring to the (a) DSSNP and (b) DSIMP. [file 12864_2025_12256_MOESM8_ESM.tif]
