# Supplementary material for: Comparison between SNP array and imputed data to estimate population structure and ROH hotspots in horse breeds
Source: BMC Genomics. 2025 Nov 29;26:1086. doi: 10.1186/s12864-025-12256-8 (PMC12670763; doi:10.1186/s12864-025-12256-8)
Supplement: Supplementary file 3 — Additional File 3. Table S2. Mean DR2 values by chromosome before and after filtering. Description: DR2 values per chromosome (ECA) are shown for the raw dataset (all imputed markers) and after applying a DR2 threshold of 0.6 to retain high-confidence markers. The last row indicates the genome-wide mean for each column. [file 12864_2025_12256_MOESM3_ESM.docx]

| **ECA** | **Raw** | **Filtered** |
| --- | --- | --- |
| 1 | 0.48 | 0.74 |
| 2 | 0.48 | 0.74 |
| 3 | 0.47 | 0.73 |
| 4 | 0.47 | 0.74 |
| 5 | 0.47 | 0.73 |
| 6 | 0.45 | 0.73 |
| 7 | 0.47 | 0.73 |
| 8 | 0.46 | 0.74 |
| 9 | 0.48 | 0.74 |
| 10 | 0.47 | 0.74 |
| 11 | 0.46 | 0.74 |
| 12 | 0.42 | 0.71 |
| 13 | 0.47 | 0.71 |
| 14 | 0.48 | 0.74 |
| 15 | 0.47 | 0.74 |
| 16 | 0.48 | 0.74 |
| 17 | 0.50 | 0.72 |
| 18 | 0.48 | 0.73 |
| 19 | 0.48 | 0.73 |
| 20 | 0.49 | 0.72 |
| 21 | 0.49 | 0.73 |
| 22 | 0.46 | 0.72 |
| 23 | 0.48 | 0.73 |
| 24 | 0.48 | 0.74 |
| 25 | 0.45 | 0.72 |
| 26 | 0.47 | 0.71 |
| 27 | 0.48 | 0.72 |
| 28 | 0.45 | 0.72 |
| 29 | 0.48 | 0.72 |
| 30 | 0.46 | 0.72 |
| 31 | 0.46 | 0.70 |
| **mean** | **0.47** | **0.73** |
