# Supplementary material for: Comparison between SNP array and imputed data to estimate population structure and ROH hotspots in horse breeds
Source: BMC Genomics. 2025 Nov 29;26:1086. doi: 10.1186/s12864-025-12256-8 (PMC12670763; doi:10.1186/s12864-025-12256-8)
Supplement: Supplementary file 9 — Additional File 9. Table S3. ROH summary statistics reported for breed and dataset. Description: The mean number of ROH per individual (NROH), the average length of ROH in Mb per individual (LROH), the population genomic inbreeding coefficient (FROH), and the breed total length covered by ROH segments in Mb (SROH). Standard deviation for each value is reported as well (SD). [file 12864_2025_12256_MOESM9_ESM.docx]

|  |  | ***N*_ROH_** | |  | ***L*_ROH_** | |  | ***S*_ROH_** | |  | ***F*_ROH_** | |
| --- | --- | --- | --- | --- | --- | --- | --- | --- | --- | --- | --- | --- |
| **Breed** |  | **DS_SNP_** | **DS_IMP_** |  | **DS_SNP_** | **DS_IMP_** |  | **DS_SNP_** | **DS_IMP_** |  | **DS_SNP_** | **DS_IMP_** |
| ORI |  | 83.44 (12.12) | 408.67 (41.05) |  | 2.81 (0.96) | 0.53 (0.20) |  | 236.86 (97.35) | 219.77 (94.09) |  | 0.10 (0.04) | 0.10 (0.04) |
| SAN |  | 45.08 (3.50) | 250.85 (22.78) |  | 3.15 (0.87) | 0.54 (0.14) |  | 141.23 (38.61) | 134.55 (38.47) |  | 0.06 (0.02) | 0.06 (0.02) |
| SIC |  | 39.29 (6.12) | 248.93 (31.83) |  | 2.52 (0.93) | 0.44 (0.14) |  | 100.61 (46.18) | 111.03 (39.97) |  | 0.04 (0.02) | 0.05 (0.02) |
| GER |  | 75.07 (13.26) | 458.87 (62.64) |  | 2.85 (0.40) | 0.50 (0.08) |  | 216.47 (55.99) | 232.99 (55.11) |  | 0.10 (0.02) | 0.10 (0.02) |
| AKT |  | 68.47 (7.95) | 380.58 (33.35) |  | 3.49 (0.64) | 0.61 (0.12) |  | 239.86 (53.83) | 230.90 (47.71) |  | 0.11 (0.02) | 0.10 (0.02) |
| ARA |  | 100.33 (10.77) | 534.96 (34.31) |  | 3.37 (1.24) | 0.61 (0.24) |  | 339.63 (139.52) | 329.05 (134.04) |  | 0.15 (0.06) | 0.14 (0.06) |
| FRA |  | 67.53 (8.95) | 348.89 (50.22) |  | 3.70 (0.78) | 0.66 (0.15) |  | 252.24 (78.43) | 232.85 (74.75) |  | 0.11 (0.03) | 0.10 (0.03) |
| ICE |  | 75.36 (9.16) | 218.64 (19.19) |  | 2.42 (0.85) | 0.59 (0.29) |  | 185.32 (85.65) | 131.28 (81.22) |  | 0.08 (0.04) | 0.06 (0.04) |
| SHE |  | 103.04 (14.48) | 313.04 (38.95) |  | 3.58 (0.91) | 0.90 (0.29) |  | 374.05 (132.21) | 287.19 (118.08) |  | 0.16 (0.06) | 0.13 (0.05) |
| QUA |  | 64.12 (10.73) | 390.02 (53.17) |  | 3.13 (0.78) | 0.54 (0.13) |  | 202.16 (65.20) | 211.35 (61.22) |  | 0.09 (0.03) | 0.09 (0.03) |
| STA |  | 86.08 (9.78) | 475.98 (58.48) |  | 4.72 (0.67) | 0.82 (0.14) |  | 408.51 (84.02) | 390.60 (78.93) |  | 0.18 (0.04) | 0.17 (0.03) |
| THO |  | 120.75 (9.53) | 679.89 (32.34) |  | 3.71 (0.27) | 0.68 (0.05) |  | 447.65 (45.88) | 462.20 (38.38) |  | 0.20 (0.02) | 0.20 (0.02) |
