# Supplementary material for: Comparison between SNP array and imputed data to estimate population structure and ROH hotspots in horse breeds
Source: BMC Genomics. 2025 Nov 29;26:1086. doi: 10.1186/s12864-025-12256-8 (PMC12670763; doi:10.1186/s12864-025-12256-8)
Supplement: Supplementary file 10 — Additional File 10. Table S4. Overlapping ROH islands between the top 0.5% of the three datasets within each breed. Description: Start and end position for each region are reported in Mb. The total number of markers within-island (M) and VEP results are reported, such as the number of markers filtered for high or moderate impact (F), as well as for the resulting gene (G) and QTL (Q) annotations. [file 12864_2025_12256_MOESM10_ESM.docx]

| **Breed** | **Chr** | **Start (Mb)** | **End (Mb)** | **DS_SNP_** | | | | **DS_IMP_** | | | | **DS_WGS_** | | | |
| --- | --- | --- | --- | --- | --- | --- | --- | --- | --- | --- | --- | --- | --- | --- | --- |
|  |  |  |  | **M** | **F** | **G** | **Q** | **M** | **F** | **G** | **Q** | **M** | **F** | **G** | **Q** |
| AKT | 3 | 120.4 | 121.3 | 34 | 0 | 0 | 0 | 2606 | 25 | 11 | 0 | 2158 | 25 | 11 | 0 |
| AKT | 8 | 35.0 | 40.5 | 73 | 0 | 0 | 0 | 9274 | 34 | 11 | 0 | 739 | 0 | 0 | 0 |
| AKT | 14 | 0.2 | 1.6 | 24 | 0 | 0 | 0 | 1458 | 3 | 2 | 0 | 3747 | 12 | 8 | 0 |
| ARA | 2 | 100.4 | 102.2 | 37 | 0 | 0 | 0 | 3087 | 7 | 3 | 0 | 943 | 2 | 1 | 0 |
| ARA | 3 | 36.2 | 39.7 | 46 | 1 | 1 | 2 | 3053 | 14 | 10 | 2 | 2759 | 9 | 4 | 0 |
| ARA | 3 | 120.7 | 121.3 | 31 | 0 | 0 | 0 | 2030 | 22 | 9 | 0 | 769 | 11 | 4 | 0 |
| ARA | 18 | 49.0 | 50.1 | 20 | 0 | 0 | 0 | 34 | 0 | 0 | 0 | 571 | 2 | 2 | 0 |
| FRA | 3 | 120.4 | 121.3 | 34 | 0 | 0 | 0 | 2774 | 20 | 10 | 0 | 2430 | 22 | 10 | 0 |
| FRA | 6 | 30.1 | 31.0 | 18 | 0 | 0 | 0 | 1910 | 13 | 3 | 0 | 222 | 1 | 1 | 0 |
| FRA | 11 | 23.1 | 32.9 | 46 | 1 | 1 | 0 | 3529 | 31 | 17 | 0 | 13571 | 74 | 30 | 0 |
| FRA | 15 | 25.7 | 28.3 | 52 | 0 | 0 | 0 | 10525 | 0 | 0 | 0 | 594 | 0 | 0 | 0 |
| GER | 2 | 100.4 | 101.1 | 16 | 0 | 0 | 0 | 195 | 0 | 0 | 0 | 455 | 0 | 0 | 0 |
| GER | 3 | 118.6 | 121.3 | 60 | 0 | 0 | 0 | 1963 | 30 | 8 | 0 | 582 | 1 | 1 | 0 |
| GER | 5 | 34.8 | 39.1 | 59 | 1 | 1 | 0 | 1866 | 27 | 9 | 0 | 554 | 3 | 3 | 0 |
| ICE | 11 | 25.3 | 32.2 | 112 | 0 | 0 | 0 | 13787 | 21 | 8 | 0 | 115 | 0 | 0 | 0 |
| ORI | 3 | 35.4 | 36.0 | 10 | 0 | 0 | 0 | 1522 | 29 | 8 | 0 | 1785 | 29 | 8 | 0 |
| ORI | 8 | 24.4 | 41.2 | 68 | 1 | 1 | 0 | 9938 | 72 | 26 | 0 | 1179 | 10 | 3 | 0 |
| QUA | 3 | 35.1 | 38.7 | 58 | 1 | 1 | 2 | 6606 | 57 | 27 | 2 | 5606 | 46 | 21 | 2 |
| QUA | 14 | 41.0 | 42.5 | 29 | 0 | 0 | 0 | 2352 | 9 | 4 | 0 | 1473 | 2 | 2 | 0 |
| QUA | 18 | 63.3 | 68.3 | 95 | 0 | 0 | 0 | 11430 | 36 | 16 | 0 | 13476 | 41 | 21 | 0 |
| SAN | 15 | 40.2 | 40.5 | 8 | 0 | 0 | 0 | 347 | 2 | 2 | 0 | 799 | 8 | 3 | 0 |
| SAN | 16 | 36.1 | 40.4 | 50 | 1 | 1 | 0 | 730 | 4 | 3 | 0 | 503 | 1 | 1 | 0 |
| SAN | 17 | 1.3 | 29.1 | 86 | 1 | 1 | 0 | 7227 | 20 | 11 | 0 | 5351 | 13 | 8 | 0 |
| SHE | 1 | 108.5 | 112.2 | 65 | 2 | 1 | 0 | 4049 | 22 | 5 | 0 | 5308 | 32 | 7 | 0 |
| SHE | 19 | 30.4 | 31.1 | 17 | 0 | 0 | 0 | 1946 | 1 | 1 | 0 | 626 | 0 | 0 | 0 |
| SIC | 7 | 51.7 | 53.6 | 39 | 1 | 1 | 0 | 5358 | 97 | 30 | 0 | 6182 | 106 | 35 | 0 |
| STA | 7 | 51.5 | 54.0 | 50 | 1 | 1 | 0 | 7576 | 141 | 48 | 0 | 2453 | 54 | 18 | 0 |
| STA | 8 | 35.3 | 47.2 | 78 | 0 | 0 | 0 | 16308 | 30 | 11 | 0 | 13094 | 16 | 8 | 0 |
| STA | 23 | 22.1 | 22.8 | 20 | 0 | 0 | 0 | 304 | 1 | 1 | 0 | 1896 | 1 | 1 | 0 |
| THO | 1 | 21.9 | 23.2 | 31 | 0 | 0 | 0 | 4122 | 0 | 0 | 0 | 3954 | 0 | 0 | 0 |
| THO | 1 | 45.3 | 47.2 | 26 | 0 | 0 | 0 | 4984 | 2 | 1 | 0 | 6027 | 2 | 1 | 0 |
| THO | 14 | 41.0 | 42.5 | 29 | 0 | 0 | 0 | 2065 | 7 | 4 | 0 | 2065 | 7 | 4 | 0 |
| THO | 17 | 20.6 | 23.4 | 51 | 0 | 0 | 0 | 7674 | 19 | 11 | 0 | 3889 | 4 | 4 | 0 |
